# The electron affinity of astatine

**DOI:** 10.1038/s41467-020-17599-2

**Published:** 2020-07-30

**Authors:** David Leimbach, Julia Karls, Yangyang Guo, Rizwan Ahmed, Jochen Ballof, Lars Bengtsson, Ferran Boix Pamies, Anastasia Borschevsky, Katerina Chrysalidis, Ephraim Eliav, Dmitry Fedorov, Valentin Fedosseev, Oliver Forstner, Nicolas Galland, Ronald Fernando Garcia Ruiz, Camilo Granados, Reinhard Heinke, Karl Johnston, Agota Koszorus, Ulli Köster, Moa K. Kristiansson, Yuan Liu, Bruce Marsh, Pavel Molkanov, Lukáš F. Pašteka, João Pedro Ramos, Eric Renault, Mikael Reponen, Annie Ringvall-Moberg, Ralf Erik Rossel, Dominik Studer, Adam Vernon, Jessica Warbinek, Jakob Welander, Klaus Wendt, Shane Wilkins, Dag Hanstorp, Sebastian Rothe

**Affiliations:** 1 CERN, Geneva, Switzerland; 2 Department of Physics, University of Gothenburg, Gothenburg, Sweden; 3 Institut für Physik, Johannes Gutenberg-Universität, Mainz, Germany; 4 Van Swinderen Institute for Particle Physics and Gravity, University of Groningen, Groningen, The Netherlands; 5 National Centre for Physics (NCP), Islamabad, Pakistan; 6 Institut für Kernchemie, Johannes Gutenberg-Universität, Mainz, Germany; 7 School of Chemistry, Tel Aviv University, Tel Aviv, Israel; 8 Petersburg Nuclear Physics Institute - NRC KI, Gatchina, Russia; 9 Institut für Optik und Quantenelektronik, Friedrich-Schiller-Universität Jena, Jena, Germany; 10 Helmholtz-Institut Jena, Jena, Germany; 11 CEISAM, Université de Nantes, CNRS, Nantes, France; 12 Massachusetts Institute of Technology, Cambridge, MA USA; 13 KU Leuven, Instituut voor Kern- en Stralingsfysica, Leuven, B-3001 Belgium; 14 Institut Laue-Langevin, Grenoble, France; 15 Department of Physics, Stockholm University, Stockholm, Sweden; 16 Physics Division, Oak Ridge National Laboratory, Oak Ridge, TN USA; 17 Department of Physical and Theoretical Chemistry & Laboratory for Advanced Materials, Faculty of Natural Sciences, Comenius University, Bratislava, Slovakia; 18 Department of Physics, University of Jyväskylä, Jyväskylä, Finland; 19 School of Physics and Astronomy, The University of Manchester, Manchester, UK; 20 Present Address: SCK CEN, Research Centre Mol, Boeretang 200, 2400 Mol, Belgium

**Keywords:** Chemical physics, Chemical physics

## Abstract

One of the most important properties influencing the chemical behavior of an element is the electron affinity (EA). Among the remaining elements with unknown EA is astatine, where one of its isotopes, ^211^At, is remarkably well suited for targeted radionuclide therapy of cancer. With the At^−^ anion being involved in many aspects of current astatine labeling protocols, the knowledge of the electron affinity of this element is of prime importance. Here we report the measured value of the EA of astatine to be 2.41578(7) eV. This result is compared to state-of-the-art relativistic quantum mechanical calculations that incorporate both the Breit and the quantum electrodynamics (QED) corrections and the electron–electron correlation effects on the highest level that can be currently achieved for many-electron systems. The developed technique of laser-photodetachment spectroscopy of radioisotopes opens the path for future EA measurements of other radioelements such as polonium, and eventually super-heavy elements.

## Abstract (web summary)

Electron affinity (EA) is a key parameter in determining the chemical behavior of the elements, but challenging to measure for unstable atoms. Here the authors succeed in measuring the EA of astatine, the heaviest naturally occurring halogen, and compare it with predictions from relativistic calculations.

## Introduction

Chemistry is all about molecule formation through the creation or destruction of chemical bonds between atoms and relies on an in-depth understanding of the stability and properties of these molecules. Most of these properties can be traced back to the molecule’s constituents, the atoms. Thus, the intrinsic characteristics of chemical elements are of crucial importance in the formation of chemical bonds. The electron affinity (EA), one of the most fundamental atomic properties, is defined as the amount of energy released when an electron is added to a neutral atom in the gas phase. Large EA values characterize electronegative atoms, i.e., atoms that tend to attract shared electrons in chemical bonds. Hence, the EA informs about the subtle mechanisms in bond making between atoms, and it also reveals information about molecular properties such as the dipole moment or the molecular stability. Contrary to neutral atoms or positive ions, the excess electron in a negative ion asymptotically sees a neutral system. As a consequence, the electron–electron correlation plays a very important role in various properties of the negative ions, and in particular in their electron affinities^1^. Hence, negative ions are excellent systems to benchmark theoretical predictions that go beyond the independent particle model.

The EA also enters into the definition of several concepts, notably the chemical potential within the purview of conceptual density functional theory (DFT), promoted by Robert G. Parr^2^, and the chemical hardness which is the core of the hard and soft acids and bases (HSAB) theory, introduced by Ralph G. Pearson in the early 1960s^3^. Robert S. Mulliken used the EA in combination with the ionization energy (IE), the minimum amount of energy required to remove an electron from an isolated neutral gaseous atom, to develop a scale for quantifying the electronegativity of the elements^4^. The usefulness of these concepts for chemists, especially in the field of reactivity, has been amply demonstrated in recent decades^5,6^.

**Fig. 1:**
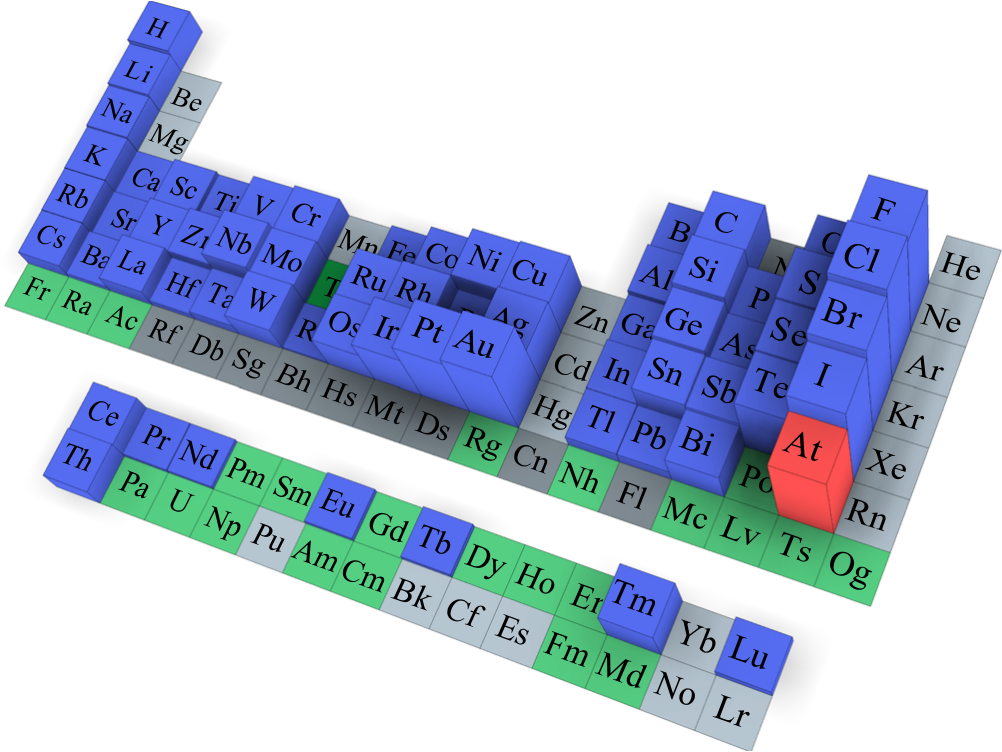
Electron affinities across the periodic table. The height corresponds to the measured value of the electron affinity of the corresponding element^7,8,67^. Astatine is highlighted in red. Blue indicates elements that are experimentally determined to have a positive EA, i.e., to form stable negative ions. Elements that are predicted to form stable negative ions but have not yet been experimentally investigated are indicated in green, while those in light gray are predicted to not form a stable negative ion, i.e., have a negative EA. Finally, elements that neither have been experimentally observed nor investigated theoretically, are indicated with dark gray.

The atomic IEs show a highly regular variation along the periodic table of elements. Starting from the lowest values at the lower left corner of the heaviest alkalines, a mostly steady trend toward higher values is observed both toward lighter elements with similar chemical behavior in one column and along rows to the right side of the chart with halogens and noble gases, with only few exceptions. Conversely, the EAs display comparably strong variations across the periodic table, as shown in Fig. 1. In this figure, some general features can be noted. For instance, the EA tends to increase as a shell is filled, then drops dramatically for elements with closed shell atomic structures, such as the noble gases which do not form stable negative ions at all, and thus have negative EAs.

The group of elements with the largest EAs are the halogens. As in most other groups of elements, no monotonic trend is observed here when progressing along the rows of the periodic table, with chlorine exhibiting the largest known EA (3.612 725(28) eV) of all elements^7,8^. The EA of the heaviest naturally occurring element in the halogen group, astatine, has not been measured to date. For this rare element, little is known of its chemistry: not only is it one of the rarest of all naturally occurring elements^9^, but the minute amounts that can be produced artificially prevent the use of conventional spectroscopic tools. For instance, while astatine was discovered in the 1940s^10,11^, it is only recently that the IE of astatine was measured through an on-line laser-ionization spectroscopy experiment at CERN-ISOLDE^12^.

However, the EA(At) has been predicted with various quantum mechanical methods^13–19^. Hence, an experimental determination of EA(At) is of fundamental interest, both to test sophisticated atomic theories and to gain bases for inferring some chemical properties of this element. The measurement of the EA(At) is also of practical interest regarding the envisaged medical applications of astatine, since certain chemical compounds containing the isotope ^211^At are currently being studied for use in cancer treatment. ^211^At, only available in nanogram quantities through synthetic production methods, is a most promising candidate for radiopharmaceutical applications via targeted alpha therapy (TAT)^20–22^, due to its favorable half-life of about 7.2 h and its cumulative *α*-particle emission yield of 100%. However, in order to successfully develop efficient radiopharmaceuticals, a better understanding of the basic chemical properties of astatine is required^23^.

The interest in the experimental determination of the EA notably lies in current labeling protocols that aim at binding astatine to tumor-targeting biomolecules: in many cases, the chemical reactions involve an aqueous astatine solution in which the astatide anion (At^−^) readily forms. In addition, a current problem for the investigated ^211^At-radiopharmaceuticals is the significant in vivo de-labeling, releasing At^−^ that could damage healthy tissues and organs of the patient^22,24,25^. The determination of the electron binding energy of the astatine anion, i.e., the EA, should help to better understand these reaction kinetics as well as the stability of involved astatine compounds.

In this paper, we present the experimental determination of the electron affinity of astatine by means of laser photodetachment threshold spectroscopy. The measured value is then compared to independent results from state-of-the-art relativistic quantum mechanical calculations carried out alongside the measurement.

## Results

### Laser photodetachment of astatine

**Fig. 2:**
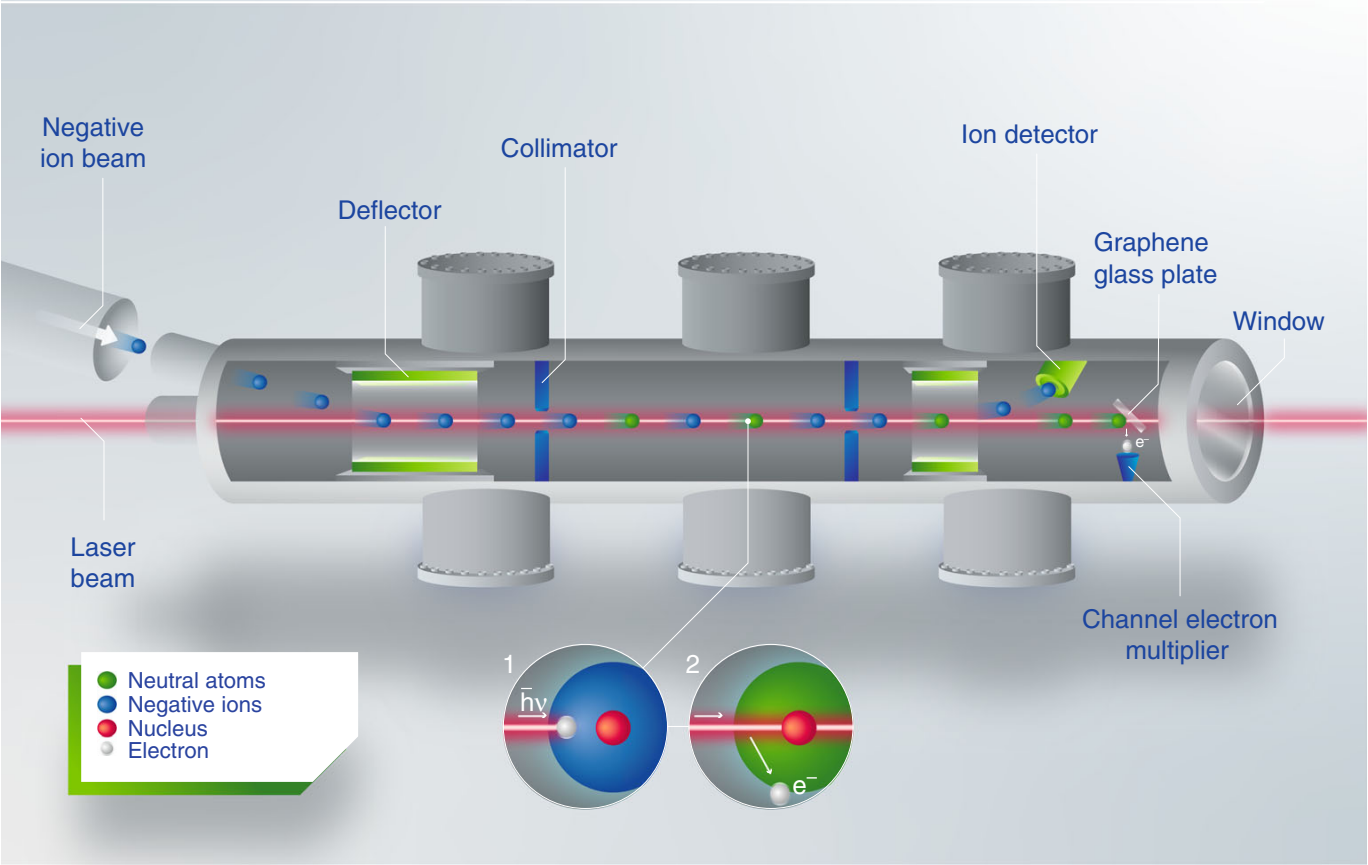
Schematic diagram of the experimental setup. From left to right: a beam of negative astatine ions (blue circles) is guided into GANDALPH^49,50^, where the ion beam is overlapped with a frequency tuneable laser beam (red line) in the interaction region in either co- or counter-propagating geometry. By absorbing a photon (Inset 1), an electron can gain enough energy to be ejected from the ion, thereby creating a neutral atom (green circles, Inset 2). After the interaction region, the charged particles are deflected into an ion detector, while neutralized atoms continue moving straight to the graphene-coated glass plate downstream and create secondary electrons (white circles), which are detected by a channel electron multiplier^51^.

Due to its scarcity and short half-life, artificial production of astatine is required to perform any experiment on this element. Thus, a laser photodetachment threshold spectrometer was coupled to an on-line isotope separator at the CERN-ISOLDE radioactive ion beam facility^26^. Here, At atoms were produced through nuclear spallation reactions of thorium nuclei, induced by a bombardment of highly energetic proton projectiles and subsequently ionized in a negative surface ion source coupled to a mass separator (further details can be found in the “Methods” section). A negative ion beam of ^211^At was extracted and superimposed with a laser beam in the Gothenburg anion detector for affinity measurements by laser photodetachment (GANDALPH) spectrometer (Fig. 2). The yield of neutral atoms produced in the photodetachment process, At^−^ + *hν* → At + e^−^, was recorded as a function of the photon energy *hν*, where *ν* is the laser frequency and *h* is Planck’s constant.

The general behavior of the photodetachment cross section *σ* just above the threshold is described by Wigner’s law^27^: *σ* = *a* + *b* ⋅*E*^*l*+1/2^, where *a* is the background level, *b* the strength of the photodetachment process, *l* the orbital angular momentum quantum number of the outgoing electron, *E* = *E*_photon_ − EA is the energy of the ejected electron and *E*_photon_ = *hν* the photon energy.

The ground state of At^−^ has a 6*p*^6 ^^1^*S*_0_ configuration. Therefore, this state shows no term, fine or hyperfine structure splitting. Further, as for all other halogen negative ions, it is the only bound state. Hence, all At^−^ ions in the ion beam are in the same quantum state, and the relatively high temperature in the ion source does not give rise to internally excited ions. In the photodetachment process, the electron is detached from a *p*-state. Close to the threshold, the angular momentum of the outgoing electron will then be *l* = 0 due to the selection rules (*Δl* = ±1) and the centrifugal barrier preventing the emission of a *d*-wave electron (*l* = 2)^1^. The ground state 6*p*^5 ^^2^*P*_3/2_ of the ^211^At atom, on the other hand, with a total angular momentum of *J* = 3/2 and nuclear spin *I* = 9/2, is split into four hyperfine levels. This splitting was recently measured with high precision by Cubiss et al.^28^. The relative strengths of these four photodetachment channels are given by the multiplicity of the final hyperfine structure levels, i.e., 2*F* + 1, where *F* = *I* + *J* is the total angular momentum of the atom, spanning from ∣*I* − *J*∣ to ∣*I* + *J*∣, i.e., 3, 4, 5, 6^29^.

The energy dependence of the cross section for photodetachment of astatine near the threshold can be described by the function

1 $$\sigma ({E}_{{\rm{photon}}})= \, 	\, a+b\mathop{\sum }\limits_{F = 3}^{6}(2F+1)\sqrt{{E}_{\rm{photon}}-({\rm{EA}}+{E}_{{\rm{hfs}},{\rm{F}}})}\\ 	\, \cdot \Theta \left({E}_{{\rm{photon}}}-({\rm{EA}}+{E}_{{\rm{hfs}},{\rm{F}}})\right)$$

where $\Theta (E-({\rm{EA}}+{E}_{{\rm{hfs}},{\rm{F}}}))$ is the Heaviside function and *E*_hfs,F_ is the energy of the hyperfine levels of the ^211^At atomic ground state, differing by less than 23 μeV between the contributing levels.

**Fig. 3:**
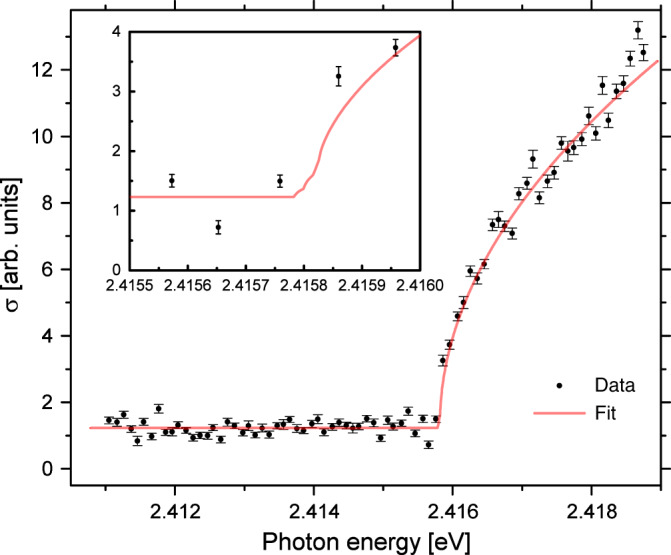
Threshold scan of the photodetachment of astatine. The neutralization cross section is measured as a function of the photon energy. The data points are the experimental measurements with one standard deviation represented by error bars, and the solid line is a fit of Eq. (1). The onset corresponds to the EA of ^211^At. The inset shows the region around threshold, where the different onsets in the fit function represent the detachment to the hyperfine levels of the groundstate of the neutral atom.

The photon energy (i.e., laser frequency) was scanned from below the threshold to well above all four hyperfine levels in the ground state of ^211^At. In total, six threshold scans were performed with laser and ion beam co- and counter-propagating, respectively. Figure 3 shows the measured neutralization cross section *σ*(*E*_photon_) as a function of the photon energy, corrected for the Doppler shift, for the sum of all threshold scans with co-propagating ion and laser beams.

The statistical error of the measurement is dominated by the laser bandwidth of 12 GHz, corresponding to 50 μeV. The contribution to the statistical uncertainty from all other effects is smaller than 0.1 μeV, as discussed further in the “Methods” section, and hence can be neglected. Systematic errors can arise due to instabilities of the ion beam energy and the determination of the photon energy. We measured the threshold for the naturally abundant ^127^I before and after the experiment on astatine, under the same experimental conditions. Those measurements differed by less than 20 μeV. This gives an estimate of a systematic error in the photon energy determination and ion beam energy stability.

Including both systematic and statistical errors, the resulting value of EA(At), determined by the geometric mean of the photodetachment thresholds measured in the co- and counter-propagating geometries, was determined to be 2.41578(7) eV.

### Theoretical calculation

Alongside the measurements, state-of-the-art calculations of the electron affinities of astatine and its lighter homolog, iodine, were carried out. The results for EA(I) served to assess the performance and the expected accuracy of the computational method. The calculations were carried out with the DIRAC15 program package^30^ using the single reference coupled-cluster approach in the framework of the Dirac-Coulomb Hamiltonian (DC-CCSD(T)), which is considered to be extremely powerful for the treatment of heavy many-electron systems. Large, saturated basis sets^31^ were used in these calculations, and extrapolation to the complete basis set limit was performed. The correction from perturbative to the full triple excitations, +ΔT, and the contribution of the perturbative quadruple excitations, +(Q), were evaluated^32^. To further improve the precision we have also accounted for the Breit interaction and the quantum electrodynamics (QED) contributions; the latter were calculated using the model Lamb shift operator (MLSO) of Shabaev et al.^33^. Further computational details can be found in the “Methods” section. The contributions of higher order excitations and Breit and QED corrections are added to the DC-CCSD(T) EAs to obtain the final values. The computational scheme outlined above was previously applied to the determination of the EA of gold, yielding an accuracy of 1.4 meV^32^. Using our knowledge of the magnitude of the various effects, we are able to set a conservative uncertainty of  ±0.016 eV on the computed values (see “Methods” section for further details). Hence, the expected value of the EA(At) from the theoretical calculations is 2.414(16) eV. The results for iodine and astatine, including the break-down of the various higher order contributions are presented in Table 1 and compared to the experimental value. The final result of the electron affinity calculation for iodine lies within 0.004 eV of the measured value of 3.059 0463(38) eV^34^.

**Table 1:** Comparison of computational and experimentally determined EAs of I and At.

Method	EA(I)/eV	EA(At)/eV
CBS-DC-CCSD(T)	3.040	2.401
+ΔT(Q)	0.008	0.007
+Breit	0.003	0.003
+QED	0.003	0.003
Final theor.	3.055(16)	2.414(16)
Exp.	3.059 0463(38)^34^	2.41578(7)

## Discussion

Over the years, many attempts were made to calculate the EA of astatine. However, the high atomic number and thus the need of refined treatments of relativity as well as the dominance of the electron correlation effects made this a challenging task. With the given uncertainties, our computed value is in excellent agreement with the experiment. This shows that careful, systematic, and as complete as possible inclusion of higher-order correlation and relativistic contributions makes it possible to achieve benchmark accuracy in atomic calculations. Hence, our measured EA(At) represents a sharp test for assessing theoretical methods used to study the chemistry of heavy and super-heavy elements. Some recent calculations, including our final theoretical value of the EA of At (labeled CBS − DC − CCSDT(Q) + Breit + QED) are compared to the experimental value in Table 2. Of particular interest is the recent multi-configurational Dirac-Hartree-Fock (MCDHF) study of Si and Fischer^13^. Including the Breit and the QED corrections and extrapolating systematically in terms of included configurations, they obtained an EA for iodine (3.0634(24) eV) in excellent agreement with the experiment. However, the analogous result for At (2.3729(46) eV) lies outside the uncertainty of our experiment. More recently, another very accurate calculation of the EA of At (and other heavy *p*-block elements) was carried out by Finney and Peterson^14^, using an approach similar to that employed in this work. They obtained an EA of 2.423(13) eV, which is in very good agreement with both the measurement and the prediction of this work. The difference between the two theoretical results is mainly due to the number of correlated electrons (all 85 in the present calculation vs. 25 in ref. ^14^), the use of the Gaunt correction (instead of Breit) in ref. ^14^ and the lack of the higher excitations in earlier work.

**Table 2:** Comparison of the present calculations of the EA of At to other theoretical approaches.

Method	EA(At)/eV	Ref.
CBS-DC-CCSDT(Q) + Breit + QED	2.414(16)	This work
MCDHF + SE corr.^a^	2.38(2)	^19^
MCDHF	2.416	^16^
DC-CCSD(T) + Breit + QED	2.412	^17^
MCDHF + Extrap. + Breit + QED^b^	2.3729(46)	^13^
CBS-DC-CCSD(T)+Gaunt+QED	2.423(13)	^14^
Experiment	2.41578(7)	This work

^a^Multiconfigurational Dirac-Fock (MCDF) results corrected using experimental data.

^b^MCDF results extrapolated to complete active space limit.

Our result of the EA of astatine, 2.41578(7) eV, indicates that among the naturally occurring halogen elements, astatine has the lowest EA. On the other hand, its EA remains larger than the measured values of all elements in the other groups of the periodic table. Therefore, this value is consistent with the tendency of halogens to complete their valence shell on gaining one extra electron. For the halogen elements, the significance of large EAs is the strong tendency to form anions in aqueous solution. A significant part of the value of the reduction potential associated with the formation of At^−^ comes from the EA. Indeed, the reduction potential in solution can be evaluated from a thermodynamic cycle^35^ involving (i) the reduction reaction in the gas phase, and (ii) the difference of Gibbs free energy of solvation between the anion and the neutral atom. The Gibbs free energy corresponding to (i) essentially comes down to the electron affinity, since the electronic partition function of At (^2^*P*_3/2_) yields an insignificant contribution and the free energy of the gas-phase free electron is almost null^35^. The contribution of (ii) is similar to (i),  ≈2.5 eV, since the solvation free energies of neutral solutes do not exceed few kcal per mol^35^ and, according to a recent estimate^36^, Δ*G*_*sol*_(At^−^) ≈ −68 kcal/mol. In addition to the EA, the IE also contributes to the determination of the nature of elemental forms of astatine in aqueous solutions: the Pourbaix (potential/pH) diagram of astatine shows coexistence of the At^+^ and At^−^ ions. Their dominance domains are governed by the redox potential E°(At^−^/At^+^)^37^, which can be evaluated as well from a thermodynamic cycle. The latter involves the difference of solvation free energy between the anion and the cation, and the formation in gas phase of astatide from the At^+^ cation^38^. The Gibbs free energy of this reaction essentially comes down to the sum of EA(At) and IE(At).

The usefulness of the EA for a better understanding of the chemistry of astatine is also shown through the deduction of the electronegativity, softness, hardness, and the electrophilicity index, which are shown in Table 3, together with the respective definitions. The list of chemical descriptors in Table 3 represents an advance over the computed data reported by Paul Geerlings and co-workers^39^ by deriving them from high precision measurements. These descriptors may be regarded as basic properties which will serve as the foundation for the design and the assessment of innovative astatine radiopharmaceuticals by theoretical and experimental chemists. The electronegativity of astatine is determined to be *χ*_M_ = 5.87 eV according to the Mulliken scale, which is significantly lower than that of hydrogen (*χ*_M_ = 7.18 eV), supporting the calculated bond polarization toward the hydrogen atom in the HAt molecule^40,41^. Hence, it must be named hydride instead of hydrogen halide as opposed to all other halogen-hydrogen molecules, where the halogen is usually the negatively charged atom. Additionally, the intermediate value of *χ*_M_(At) between the electronegativities reported for boron (4.29 eV) and carbon (6.27 eV) atoms, allows us to anticipate different polarizations for At-B and At-C bonds. This simple analysis is of high relevance to the use of astatine in nuclear medicine. The applications in TAT are currently hindered by the rapid de-astatination of carrier-targeting agents that occurs in vivo. In radiosynthetic protocols^24,25^, most reported biomolecules of interest have been labeled with ^211^At by formation of At-C or At-B bonds. The greater stability observed in vivo for the At-B bonds could be related to the polarization of those bonds toward the astatine atom^42^. The electrophilicity index is particularly relevant in view of the currently prevalent approach for the ^211^At-radiolabelling, which is supposed to bind astatine to carrier molecules through an electrophilic substitution^24,25^. In addition, recent studies have illustrated how the electrophilicity of the astatine atom modulates the ability of astatinated compounds to form stabilizing molecular interactions known as halogen bonds^43,44^. The moderate value of hardness, *η*(At) = 3.45 eV, is consistent with the observed high affinity of astatine in direct attachment experiments with proteins bearing soft sulfur donor groups^45^, according to the hard and soft (Lewis) acids and bases (HSAB) theory (*η*(S) = 4.14 eV for the S atom^46^).

**Table 3:** Values and definitions of properties of astatine derived from the EA and IE.

Property	Definition	Value
Electron affinity	EA	2.41578(7) eV
Ionization energy	IE	9.31751(8) eV^12^
Electronegativity	${\chi }_{{\rm{M}}}=\frac{{\rm{IE}}+{\rm{EA}}}{2}$	5.86665(7) eV
Hardness	$\eta =\frac{{\rm{IE}}-{\rm{EA}}}{2}$	3.45087(7) eV
Softness	$S=\frac{1}{2\eta }$	0.14489(2) eV^−1^
Electrophilicity	$\omega =\frac{{\chi }_{{\rm{M}}}^{2}}{2\eta }$	4.98680(16) eV

In conclusion, we have carried out a measurement of the electron affinity of astatine and determined it to be EA(At) = 2.41578(7) eV. In addition, relativistic calculations carried out alongside the experiment are in excellent agreement with the experimental results, supporting the reliability and accuracy of the theoretical description. The EA of astatine is thus an excellent case for benchmarking theoretical models in atomic physics since it requires a full relativistic many-body treatment that also includes Breit and QED effects. These theoretical models can then be applied to the chemistry of elements heavier than astatine.

By combining the present result with the recent measurement of the ionization energy of astatine^12^, we were able to determine several fundamental chemical properties of this element: namely the electronegativity, softness, hardness, and electrophilicity. For instance, it can be concluded from our results, that in the astatine-hydrogen molecule, contrary to all other hydrogen halides, the hydrogen atom is more electronegative than the halogen element. Hence, according to chemical nomenclature this molecule should be called astatine hydride rather than hydrogen astatide.

As ^211^At is a promising candidate for TAT, these properties have direct implications for its use in cancer treatments. Most of ^211^At-radiopharmaceuticals suffer from in vivo release of astatide (At^−^) and the development of radiosynthetic procedures so far is severely hampered by the limited knowledge of the chemical properties of this element. Hence, accurate values of electron affinity, electronegativity, softness and electrophilicity, all issued from experiments, open up several perspectives that chemists and radiopharmacists can take advantage to understand the stability of astatine-labeled compounds. Considering that oxidative mechanisms may be responsible for in vivo dehalogenation^22^, the expected polarization toward the carbon atom, at the expense of astatine, has notably been highlighted for At-C chemical bonds. Potential impacts on the development of more efficient radio-labeling protocols cannot be ruled out.

Finally, the on-line technique presented in this work enables further EA measurements of artificially produced, short-lived radioactive elements with high precision. At ISOLDE, isotopes with half-lifes down to the millisecond range can be studied, which is limited by the time needed to extract and transport the ions from the target unit to the GANDALPH detector. However, studies of the short-lived elements which are normally produced with lower yields will require an improved detection system. Currently, a new detector based on the multi reflection time-of-flight (MR-TOF) technique is being developed, where each produced ion will be allowed to interact with the laser light for a much longer time. Furthermore, the excellent performance of the relativistic coupled-cluster method for astatine, and the robust scheme for estimation of theoretical uncertainties demonstrates the strong predictive power of this method. This will become extremely important for the superheavy elements where the low production rates and short lifetimes will necessitate reliable theoretical support for the success of the measurements and interpretation of results.

## Methods

### Negative astatine ions

**Fig. 4:**
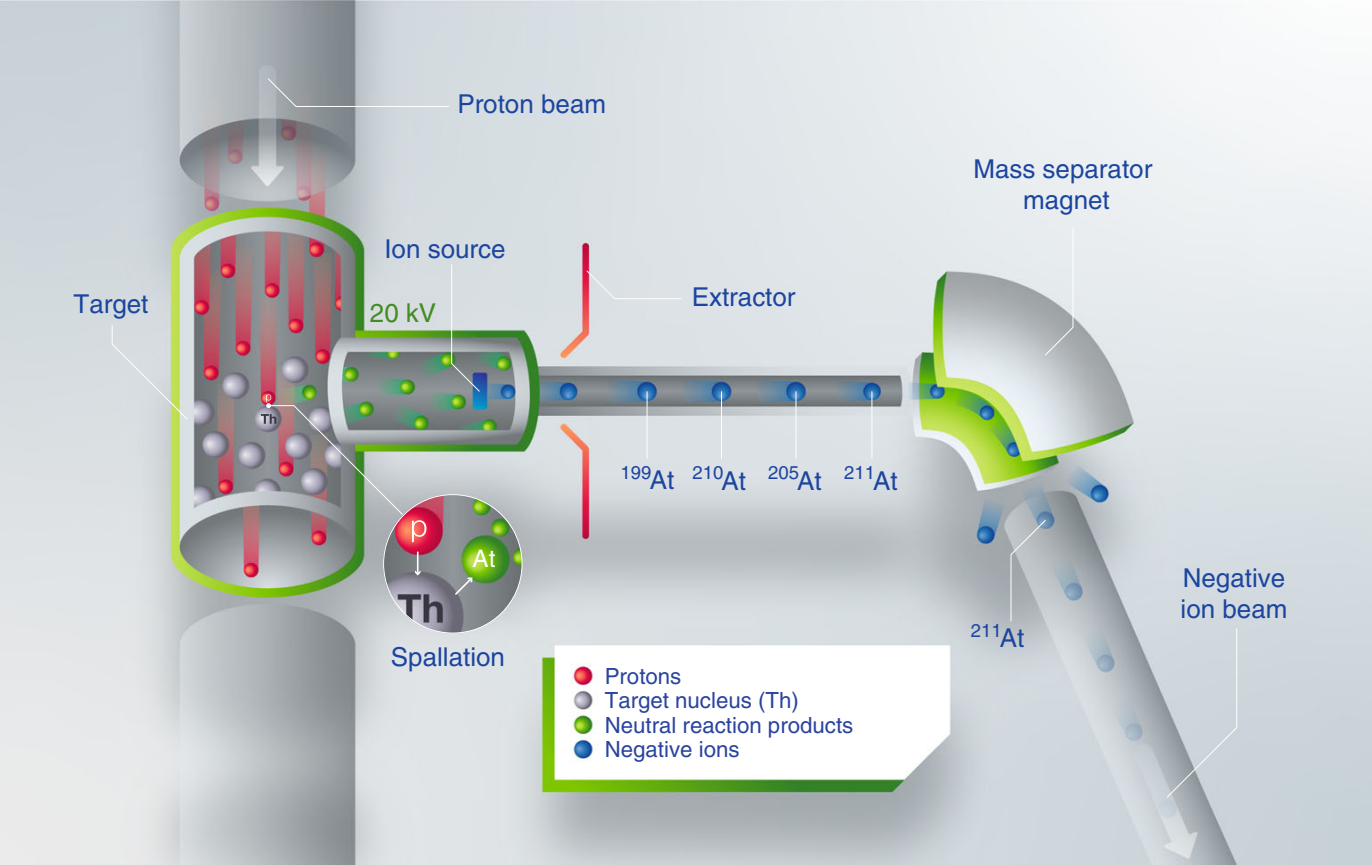
Production of a negative astatine ion beam. Astatine atoms (green circles) are created in a spallation reaction of thorium (white circles) with 1.4 GeV protons (red circles). Subsequently, the atoms are negatively ionized and extracted as a mono-energetic beam (blue circles) with an energy of 20 keV. The ^211^At isotopes are then mass separated with an electromagnetic mass separator and directed to the GANDALPH spectrometer.

Astatine isotopes were produced at the CERN-ISOLDE radioactive ion beam facility^26^. A proton beam with an energy of 1.4 GeV provided by the CERN accelerator complex impinged onto a thick Th/Ta mixed foil target, which was resistively heated to 1450 °C. A schematic view of this process is given in Fig. 4. The reaction products diffused from the target matrix and effused into an ISOLDE-MK4 negative surface ion source^47^, comprised of a hot tantalum transfer tube and a LaB_6_ surface ionizer pellet heated to 1300 °C.

Thermionic electrons emitted from the hot LaB_6_ surface were deflected with a 0.04 T permanent magnetic field and absorbed in a dedicated electron collector. Negative ions produced on the hot surface were accelerated across a 20 kV extraction potential and thereafter directed through the ISOLDE general purpose mass separator magnet (GPS). The resolution of the mass separator was sufficient to select a single isobar, which in our case was ^211^At.

In order to ensure stable astatine beam intensity throughout the experiments, the pulsed proton impact on the target was distributed equidistant in time with an average current of about 1.8 μA. An average ion current of about 600 fA (3.75 × 10^6^ particles per s) of ^211^At^−^ was measured using a Faraday cup (FC) inserted in the beam path just before the experimental chamber.

### Laser setup

The phototodetachment experiment was performed using a part of the ISOLDE RILIS (Resonance Ionization Laser Ion Source) laser system which normally serves for production of positively charged ion beams^48^. In particular, laser radiation tuneable in the range of 2.384 eV to 2.53 eV (490 nm to 520 nm) was generated by a commercial dye laser (Credo Dye, Sirah Laser-und Plasmatechnik GmbH) operated with an ethanol solution of Coumarin 503 dye. This laser was pumped by the third harmonic output (3.4925 eV) of a pulsed Nd:YAG INNOSLAB laser (CX16III-OE, EdgeWave GmbH) with a 10 kHz pulse repetition rate. Beam delivering optics comprising a set of lenses and mirrors were installed to transport the dye laser beam from the RILIS laboratory to the GANDALPH photodetachment apparatus over a distance of about 15 m. In the laser-ion beam interaction region, the laser power was in the range of 20–30 mW. Typical values of the spectral bandwidth and pulse duration emitted by the dye laser were 12 GHz and 7 ns, respectively. The laser radiation frequency was scanned in the range of 2.4110 eV–2.4301 eV (510  nm–514 nm), determined according to earlier theoretical predictions of the EA(At)^17^. The photon energy of the laser radiation was measured continuously using a wavelength meter (WS7, HighFinesse/Ångstrom).

### Collinear laser photodetachment threshold spectroscopy with GANDALPH

The GANDALPH detector, illustrated in Fig. 2, is a detector designed for measurements of the EA of radioactive elements by collinear laser photodetachment^49,50^. Electrostatic beam steering and ion optical elements are used to superimpose a continuous negative ion beam with a pulsed laser beam within the interaction region of the GANDALPH spectrometer, which is defined by two apertures of 6.0 mm diameter placed 500 mm apart. The experimental layout allows both co- and counter-propagating geometries for laser and ion beams respectively.

When a negative ion absorbs a photon of sufficient energy, its extra electron can be detached, creating a fast moving neutral atom. The Doppler shift resulting from the velocity of the ion beam in reference to the detector and laser rest frame, can be eliminated to all orders by taking the geometric mean of the measurements which are recorded in co- and counter-propagating geometry of the laser and the ion beam, respectively.

Subsequent to the interaction region, all charged particles are deflected into either a FC or a channel electron multiplier (CEM,(Channeltron XP-2334, DeTech)), allowing for continuous monitoring of the ion beam intensity. Neutral atoms proceed forward and impinge on a target made of a graphene-coated quartz plate^49,51,52^.

Secondary electrons created by the impact of the neutral atoms on the target are extracted and deflected into a second CEM, placed off-axis and biased with a potential of 2.2 kV. The signal originating from the CEM is amplified with a pulse amplifier (TA2000B-2, FAST ComTec GmbH) by a factor of 40 and fed into a gated photon counter (SRS400, Stanford Research Systems) connected to a computer. A data acquisition cycle is triggered by the signal of the photoelectrons resulting from the laser pulse impinging on the glass plate target. Due to the time of flight from the interaction region to the glass plate, the neutral atoms created in the photodetachment process arrive in the time window 2.2 μs–4.9 μs after the photon impact. Hence, the data acquisition is set to record the signal within this time window after the trigger. Background measurements are performed simultaneously by setting a second measurement gate of the same width but delayed by 12 μs after the laser pulse.

We estimate the transmission from the FC positioned in the chamber in front of GANDALPH to the detectors placed after the interaction region to be ≈1%, calculated from the initial intensity of 600 fA before the setup and the ion velocity (135,000 m/s), derived from ${E}_{{\rm{kin}}}=\frac{1}{2}m{v}^{2}$. This means that there were only 0.1 ions on average in the interaction region. Nevertheless, we observed a photodetachment signal as high as 50 counts/s of neutralized ^211^At in the GANDALPH beam-line when the photon energy was tuned well above the photodetachment threshold. Under these conditions, the combined neutralization and detection efficiency for an ion in the interaction region, which was illuminated by the 10 kHz repetition rate pulsed laser light, was 5%.

### Accuracy of EA measurements

The uncertainty in our experiment is dominated by the laser bandwidth of 12 GHz, corresponding to 50 μeV^48^. In addition, there are several minor effects contributing to the uncertainty: for a LaB_6_ surface ionizer, as used in this experiment, the energy spread has been determined to be of the order of 0.55 eV^53^. This implies a velocity spread of the ions which is compressed due to the acceleration over a high potential in the subsequent ion beam extraction process^54^.

The compressed velocity spread of the ions is given by the expression $\Delta v=\Delta W/\sqrt{2mW}$, where *m* is the ion mass, Δ*W* the energy spread of the ions and *W* the kinetic energy of the ion beam^55^. The velocity spread of the ion beam can be converted to a spread of the frequency of the laser light of Δ$\nu$ = Δ*v*/*λ* seen by the ions. This results in a frequency Doppler broadening of only a few MHz in the fast ion beam. The divergence of the ion and laser beams and the interaction time will also contribute to the broadening. However, this accumulates to uncertainties of less than 10 MHz. Consequently, the uncertainties arising from these minor effects could be ignored and only the laser bandwidth of 12 GHz needs to be considered.

In addition to these statistical errors, some systematic uncertainties arise: the Doppler shift due to the velocity difference of ions and photons is very large, but it can, as described above, be eliminated to all orders by performing the experiment with both co- and counter-propagating laser and ion beams and calculating the geometric mean to determine the Doppler-free threshold. Hence, the Doppler shift does not contribute to the uncertainty of the result, barring slight potential angle misalignment of maximum 24 mrad as defined by the apertures. However, uncertainties of the ion beam energy and the wavelength calibration could potentially affect the results. Such drifts were estimated to be smaller than 20 μeV by comparing two reference scans on stable ^127^I which were performed with the same setup before and after the measurements on astatine.

### Computational details

To achieve an optimal accuracy in the DC-CCSD(T) calculations, all electrons of iodine and astatine were correlated, and all virtual orbitals with energies below 2000 a.u. were included in the virtual space. Fully uncontracted correlation-consistent all-electron relativistic basis sets of Dyall (dyall.aeXz) were used^31^. In order to obtain accurate results for the EA, high quality description of the region removed from the nucleus (that will contain the added electron) is important. We have thus augmented the basis sets with two diffuse functions for each symmetry block. Finally, we performed an extrapolation to the complete basis set (CBS) limit, using the scheme of Halkier et al.^56^ for the DHF values and the CBS(34)^57^ scheme for the correlation contribution. In the DC-CCSD(T) calculations, the finite size of the nucleus was taken into account and modeled by a Gaussian charge distribution^58^ within the DIRAC15 program package.

Full triple and perturbative quadruple (Q) contributions were calculated in a limited correlation space with the valence 6*s* and 6*p* electrons and a virtual orbital energy cutoff of 30 atomic units. It has been previously demonstrated that higher-order correlation is dominated by the valence contributions^32^, and thus this correlation space was deemed sufficient. The valence vXz basis sets of Dyall^31^ were used, and extrapolated to the CBS limit as above. These calculations were performed using the program package MRCC^59–63^ linked to DIRAC15. Full Q contributions evaluated at the v2z level were below 1 meV for both systems and were thus omitted.

Due to the non-instantaneous interaction between particles being limited by the speed of light in the relativistic framework, a correction to the two-electron part of *H*_D*C*_ is added, in the form of the zero-frequency Breit interaction calculated within the Fock-space coupled-cluster approach (DCB-FSCC), using the Tel Aviv atomic computational package^64^. To account for the QED corrections, we applied the model Lamb shift operator (MLSO) of Shabaev and co-workers^33^ to the atomic no-virtual-pair many-body DCB Hamiltonian. This model Hamiltonian uses the Uehling potential and an approximate Wichmann–Kroll term for the vacuum polarization (VP) potential^65^ as well as local and non-local operators for the self-energy (SE), the cross terms (SEVP) and the higher-order QED terms^66^. The implementation of the MLSO formalism in the Tel Aviv atomic computational package allows us to obtain the VP and SE contributions beyond the usual mean-field level, namely at the DCB-FSCC level.

The three remaining known sources of error in these calculations are the basis set incompleteness, the neglect of even higher excitations beyond (Q), and the higher-order QED contributions. The first of these is the largest. We have extrapolated our results to the complete basis set limit, and as the associated error, we take half the difference between the CBS result and the doubly augmented ae4z (d-aug-ae4z) basis set value which is 0.015 eV. We assume that the effect of the higher excitations should not exceed the (Q) contribution of 0.004 eV, and that the error due to the incomplete treatment of the QED effects is not larger than the vacuum polarization and the self energy contributions of 0.003 eV. Combining the above sources of error and assuming them to be independent (and assuming no uncertainties beyond those discussed above), the total conservative uncertainty estimate on the calculated EA of At is 0.016 eV, dominated by the basis set effects. It should be noted that the hyperfine structure of the neutral atom was not considered in the calculations. However, the correction due to the hyperfine structure would be of the order of 10 μeV, in comparison with the estimated uncertainty in the calculation of 0.016 eV. Hence, correcting for the hyperfine structure would not change the given theoretical value and can therefore be neglected.

## Supplementary information

Supplementary Information

## Data Availability

The datasets generated and/or analyzed during the current study are available at 10.5281/zenodo.3924371.

## Code availability

The code used to analyze the datasets of the current study are available at 10.5281/zenodo.3924371.
