# Supplementary material for: The electron affinity of astatine
Source: Nat Commun. 2020 Jul 30;11:3824. doi: 10.1038/s41467-020-17599-2 (PMC7393155; doi:10.1038/s41467-020-17599-2)
Supplement: Supplementary file 1 — Supplementary Information [file 41467_2020_17599_MOESM1_ESM.pdf]

Reviewers' Comments:

Reviewer #1:
Remarks to the Author:
The manuscript reports the first measurement of the electron affinity of astatine, using the 211 isotope with a half-life of 7.2 h, hence a filling a gap in our basic knowledge of the elements of the periodic table. This clearly justifies publication in Nature Communications. Unfortunately, the manuscript in its present form contains some glitches that needs the be taken care of before publication is possible. My detailed comments are:
1) The measurement is supported by high-level calculations. In the computational details we learn that all electrons were correlated and that there was no truncation of the virtual space; at least the former is shown to be very important for high quality results. We also learn that fully uncontracted correlation-consistent all-electron relativistic basis sets of Dyall were used, but which flavor ? The DIRAC software comes with several basis set libraries, in part distinguished by what correlation functions were included. In principle, then, the dyall.aeXz sets should be used, and since the anion is considered, the augmented version dyall.aaeXz, possibly with even further augmentation, as is indeed the case here. The authors have to specify what precisely what basis set was used in order for a proper assessment of the quality of their calculations can be made.
2) In the introduction we read "Since the attraction from the nucleus is efficiently screened by the core electrons, the value of the EA is mainly determined by electron-electron correlation". The reported EA is 2.41578 eV. A simple Delta-SCF calculation using the atomic GRASP code gives 1.519 eV, or 63 % of the EA. The statement in the manuscript hence does not seem correct. The argument is based on screening of the nuclear charge by the other electrons, which is a useful, but somewhat hand-waving argument. An attempt to quantify it is by looking at the radial expectation value of the orbital under consideration and find the nuclear charge that reproduces this value for a one-electron system. In the present case we find that the effective unclear charge of the 6p3/2 orbital is around 17, which is far from the nuclear charge of 85, but not zero. I think you will have to sharpen your statement. In passing I note that Koopmans' theorem (using the HOMO of the anion), which lives on error cancellation between orbital relaxation and electron correlation, gives 2.306 eV.
3) We also read that the ionization energy is essentially determined by the Coulomb attraction between the electrons and the nucleus. Are you sure ? Is there a reference ? Apparently as a consequence the IEs show "a specific and well understood variation along the periodic table of elements". Furthermore: "the EAs display comparably strong irregularities and variations across the periodic table". Looking at your figure 1 (or the similar figure found at https://www.webelements.com/periodicity/electron_affinity/) I would say that one sees a number of features that are well understood: i) The EA typically decreases down a column. A striking exception is the late transition metals, and this can be understood from the lanthanide contraction (see e.g. Ohanession et al, JACS 112 (1990) 7179). ii) The EA drops dramatically and even vanishes for closed shells due to their stability, but also for half-filled shells, that are also particularly stable. iii) Apart from this, the EA is seen to increase as we fill a shell since along a row the increased nuclear charge tends to win over the added electron, hence causing a stabilization of orbitals. I would perhaps sharpen this discussion as well.
4) The measurement is based on the developed technique of laser-photodetachment. We learn that the ions are generated at high temperature, so an obvious thing we would like to know is to what extent they are cooled upon entering the spectrometer. Are we guaranteed that they are all in the ground state ? I believe the experimental setup is sound, but I feel this point should be sharpened.
5) The manuscript concludes by stating that "the on-line technique presented in this work enables further EA measurements of artificially produced, short-lived radioactive elements with high precision" and "paving the way for both experimental and theoretical studies of superheavy elements". These are very interesting and promising perspectives indeed. However, in the present work the half-life of the species was around seven hours. Maybe the authors could discuss approximately what half-lives would be too short for the present setup ?
6) On page 6 we read that the astatine anion "is a closed shell system with no internal structure".

I think that the authors want to say that it does not contribute to any splittings of spectral lines. However, it sounds a bit awkward to say that such a multielectron atom has no internal structure and I would recommend to reformulate.
7) The computational machinery is essentially the same as reported by many of the same theoretician in PRL 118, 023002 (2017), but contrary to the EA/IE of the neutral gold atom the effect of adding higher excitations (T and Q) are quite modest, so that statement in the abstract "require incorporation of the electron-electron effects on the highest possible level" is perhaps a bit exaggerated. Please sharpen. Clearly very high-level calculations were carried out (provided a good choice of basis set was made).
8) In the conclusion: "in contrary to" -> "contrary to"
9) In computational details: "is deemed" -> "was deemed"
10) The title of ref. 56 is very wrong ! There are also minor corrections to be done in the titles of refs. 7, 10 and 54.

Reviewer #2:
Remarks to the Author:
This manuscript by Leimbach et al. reports the first experimental determination of the electron affinity (EA) of astatine using an elegant approach at CERN-ISOLDE. As the authors point out, EA is among the most fundamental atomic properties, and it can afford insights into elemental chemistry. The experimental result also provides a basis to assess computational results and methodologies. A new computed EA(At) is presented and compared with both the experimental and previous computational results. The impressive success of the experiment provides a platform for devising approaches to determine EAs of even heavier and more scarce elements. Both the experimental approach and the resulting determination of EA(At) merit publication in Nature Communications. The manuscript is generally well written and clear. I offer below some comments that need to be addressed by the authors when preparing a revision.
1) Page 2, line 39: Change "electron-electron correlation effects on the highest possible level" to "electron-electron correlation effects on the currently highest practical level". Let's hope that in future even more accurate approaches are developed.
2) Page 3, line 55: Provide a reference for the statement that "EA is mainly determined by electron-electron correlation". Given that EA is generally higher for small atoms it would be helpful to clarify the seeming implication here that electron-nucleus interactions have only minor effects on EA.
3) Page 3, line 75: Change "largest EA" to "largest known EA".
4) Page 4, line 85: The EA of an element does not provide "precise knowledge about the chemical properties". At best it provides an imprecise basis to infer some chemical properties. Actual chemical properties of the likes of At are instead provided by approaches such as the elegant work described in ref. 38. Several of my following comments focus on the overemphasis on purported accurate derivation of condensed phase chemical behavior from EA, IE, and derived atomic properties.
5) Page 5, line 98: The EA is neither required nor sufficient to "describe these reaction kinetics" or "the stability of astatine compounds". Such claims as to the utility of EA for predicting or understanding complex condensed phase phenomena must be tempered.
6) Page 7, Figure 3: Show an expansion of the plot in the threshold region, 2.4155-2.4160 eV, with the assigned threshold clearly indicated, this to allow the reader to directly assess the accuracy of the determination. Also specify the energy separation between the last point before and the first point after the photodissociation onset; this energy resolution of the experiment is important.
7) Page 8, line 132: It is interesting that the isotope 127I is specified. Were the computations for 211At? Is there a significant isotope variation of EA and derived atomic properties?
8) Page 9, Discussion Section: Table 3 and discussion of those values belongs here rather than in the Methods section. In the first paragraph of this section it is noted that "our computed value is in excellent agreement with experiment" and that the employed computational approach is

“necessary for achieving benchmark accuracy”. However, it is apparent from Table 3 that other computed values are also in very good agreement with the new experimental value, which does not per se validate those computational methodologies. The excellent agreement for the value from ref. 15, for example, neither certifies the MCDHF method nor its application to other systems. The current agreement does not validate the employed computational approach beyond its application to EA(At).
9) Although the rationale on page 17 for the assigned uncertainty to the computed EA(At) is reasonable, any such assignments must be qualified by the acknowledgement of possible unrecognized errors in the computed values. The unreasonably (and evidently incorrect) small uncertainty assigned to the ref. 12 value in Table 3 clearly illustrates this common mistake that should not be repeated here despite the good agreement between the current experiment and theory.
10) Page 10, line 167: The sentence beginning with “In fact, the redox potential…” needs to be clearly and properly referenced and justified.
11) Page 10, line 172: Although it may be valid that E°(At-/At+) “directly depends on the EA(At) and IE(At)” it should be emphasized that other factors are important. The role of solvation of At- and At+ are key aspects, as discussed in ref. 39.
12) Page 11, Table 2: The key result of the work is the first experimental value for EA(At). The new computed value is so similar to all previous computed values (see Table 3) that it really provides no new chemical insights. The derived values in Table 2 are interesting but there is no significant revision in the predicted chemistry of At. The values from Geerlings and co-workers (ref. 47) should be included in Table 2 as they are actually remarkably good and they serve to predict essential chemical properties of At. For example, revision of the electronegativity of At from 5.74 eV in ref. 47 to 5.86665(7) eV here does not significantly revise the predicted chemistry of At; nor does it significantly revise our understanding of the chemistry of At.
13) Page 12, line 216: The sentence beginning “Hence, the new information…” should be deleted or revised. There is no new information about astatine’s chemical properties that will guide development of innovative radio-labelling protocols. Instead there is some refinement in parameters that might have been employed to roughly predict some aspects of the chemical behavior of At, if this behavior were not already known.
14) Page 12, line 219: The point in the sentence beginning with “Finally,…” is a very positive result of this work. The manuscript would benefit from elaboration as to how the technique might be modified for application to much lower ion abundances such as would be the case for superheavy elements.
15) Page 12, line 220: Regarding the sentence beginning with “Furthermore…”, favorable results for At (Z = 85) do not demonstrate nor pave the way for theoretical studies of superheavy elements (Z ≥ 104). Emphasize here and elsewhere the impressive experimental “paving”.

Reviewer #3:
Remarks to the Author:
Report on "The electron affinity of astatine”, ms. NCOMMS-20-05667-T by David Leimbach et al.
This manuscript reports on the first experimental measurement of the electron affinity of astatine, with a comparison with ab initio calculations.
The work seems basically sound and it will very likely make a valuable contribution to our knowledge of atomic electron affinities. A few details however call for additional explanations or amendments.
Figure 1 should be updated taking the publication of a recent measurement of the electron affinities of Pr, Nd and Tb into account (the electron affinity of Pr had been in principle already known but the measurement led to a substantial reduction of its value): X. Fu et al., Phys. Rev. A 101 (2020) 022502.
On page 6 I do not understand why so many electrons appear in the zoomed parts of figure 2, especially zoom number 2: why should photodetachment make two electrons appear?
Page 7, “Heaviside” is Oliver Heaviside’s name and thus deserves an initial capital letter.

On page 8 I wish we were given the results of the “reference measurements” performed on 127I to consolidate the estimation of the uncertainty. The result of the calculation of the electron affinity of iodine is given explicitly; why not show us the measured electron affinity?

It is not clear whether the described theoretical calculation of the electron affinity does include the hyperfine splitting of the neutral atom. My impression, even though the authors tell the nuclear volume is taken into account, is that the splitting is not considered. As a consequence, the final value of the calculated electron affinity is probably the hyperfine-averaged value, while the experimental value refers to the actual ground level of the neutral atom, i.e. the lowest hyperfine sublevel. The difference, if I translate Cubiss et al.’s parameters properly, is a bit more than 10 µeV. This is much smaller than the uncertainty of the calculated value, but this is enough to change the last digit of the experimental value, if one wants to translate it into a hyperfine-averaged one. The question of whether the hyperfine structure has been included in the calculation should be made clear anyway and, if comparisons are made, those comparisons must be made between identically defined quantities.

On page 9, the authors tell us of a “meV accuracy”, but the quoted reference actually tells of a 1.4 meV difference between theory and experiment, which is slightly larger. The accuracy reached by previous calculations on gold should be described in a more precise way, without overstating the accuracy reached by reference 35.

On page 10, the authors wonder that At has the lowest electron affinity among the halogens. This was, however, quite predictable as a fine structure effect. Even though the fine structure of the ground term of astatine has remained, as far as I know, unmeasured, one can rely on the observation of a very large fine structure in iodine (about 0.94 eV) to predict that the fine structure splitting of neutral astatine, the larger Z the greater the fine structure, will be even greater than 1 eV. As the negative ion At-, due to its complete outer shell, has no fine structure, huge fine structure lowering of the atom’s ground level has the straightforward effect of making the electron affinity of At substantially smaller than the electron affinities of lighter atoms in the same column. A similar effect has already been observed in Pb, which is not that different from the lighter atoms of its column IV B, but owes its much smaller electron affinity to a huge fine structure splitting of the neutral ground level (and, again, absence of ground-term fine structure splitting, which, for the anions of column IV B, is due to the fact that this ground term is an S one), see D. Bresteau et al., J. Phys. B: At. Mol. Opt. Phys. 52 (2019) 065001. These considerations would deserve being alluded to, especially when one compares the electron affinity of astatine to the electron affinities of the other atoms of column VII B, and of course made more precise if the fine structure of astatine happens to have been measured somewhere, which I have not been aware of.

Response to reviewer 1

April 2020

Reviewer 1

The manuscript reports the first measurement of the electron affinity of astatine, using the 211 isotope with a half-life of 7.2 h, hence filling a gap in our basic knowledge of the elements of the periodic table. This clearly justifies publication in Nature Communications. Unfortunately, the manuscript in its present form contains some glitches that needs to be taken care of before publication is possible. My detailed comments are:

Author response:

We thank all the reviewers for the support, the time spent on the comments and consequently the valuable suggestions leading to the improvement of the manuscript.

We have addressed the issues raised point by point in the text below, hopefully resolving the glitches indicated by the reviewer 1.

Additionally, the changes suggested by other reviewers are highlighted in the revised version of the manuscript.

1

The measurement is supported by high-level calculations. In the computational details we learn that all electrons were correlated and that there was no truncation of the virtual space; at least the former is shown to be very important for high quality results.
We also learn that fully uncontracted correlation-consistent all-electron relativistic basis sets of Dyall were used, but which flavor?
The DIRAC software comes with several basis set libraries, in part distinguished by what correlation functions were included. In principle, then, the dyall.aeXz sets should be used, and since the anion is considered, the augmented version dyall.aaeXz, possibly with even further augmentation, as is indeed the case here. The authors have to specify precisely what basis set was used in order for a proper assessment of the quality of their calculations can be made.

Response to the Reviewer:

We now specify in the text that the dyall.aeXz basis sets were used.

Suggested changes to the manuscript:

The following sentence (page 18, line 364):

"To achieve an optimal accuracy in the DC-CCSD(T) calculations, all electrons of iodine and astatine were correlated, and all virtual orbitals with energies below 2000 a.u. were included in the virtual space. Fully uncontracted correlation-consistent all-electron relativistic basis sets of Dyall were used [#]."

was modified to (changes highlighted in red):

"To achieve an optimal accuracy in the DC-CCSD(T) calculations, all electrons of iodine and astatine were correlated, and all virtual orbitals with energies below 2000 a.u. were included in the virtual space. Fully uncontracted correlation-consistent all-electron relativistic basis sets of Dyall (dyall.aeXz) were used [#]."

2

In the introduction we read "Since the attraction from the nucleus is efficiently screened by the core electrons, the value of the EA is mainly determined by electron-electron correlation". The reported EA is 2.41578 eV. A simple Delta-SCF calculation using the atomic GRASP code gives 1.519 eV, or 63 % of the EA. The statement in the manuscript hence does not seem correct.
The argument is based on screening of the nuclear charge by the other electrons, which is a useful, but somewhat hand-waving argument. An attempt to quantify it is by looking at the radial expectation value of the orbital under consideration and find the nuclear charge that reproduces this value for a one-electron system. In the present case we find that the effective nuclear charge of the 6p3/2 orbital is around 17, which is far from the nuclear charge of 85, but not zero. I think you will have to sharpen your statement. In passing I note that Koopmans' theorem (using the HOMO of the anion), which lives on error cancellation between orbital relaxation and electron correlation, gives 2.306 eV.

Response to the Reviewer:

We agree that the statement "the value of the EA is mainly determined by electron-electron correlation" is not correct. We would like to express that, due to the lack of long range Coulomb interaction, the electron-electron correlation is of greater importance in negative ions as compared to atoms or positive ions. We have therefore rephrased the sentence to correct our statement, and added a reference to a review on negative ions.

Suggested changes to the manuscript:

The sentence (page 3, line 55):

"Since the attraction from the nucleus is efficiently screened by the core electrons, the value of the EA is mainly determined by electron-electron correlation"

has been replaced by:

"Due to the lack of the long range Coloumb interaction, the electron-electron correlation is of greater importance in negative ions as compared with atoms or positive ions [Pegg2004]."

3

We also read that the ionization energy is essentially determined by the Coulomb attraction between the electrons and the nucleus. Are you sure ? Is there a reference? Apparently, as a consequence, the IEs show "a specific and well understood variation along the periodic table of elements". Furthermore: "the EAs display comparably strong irregularities and variations across the periodic table". Looking at your figure 1 (or the similar figure found at webelements.com) I would say that one sees a number of features that are well understood: i) The EA typically decreases down a column. A striking exception is the late transition metals, and this can be understood from the lanthanide contraction (see e.g. Ohanession et al, JACS 112 (1990) 7179).
5

ii) The EA drops dramatically and even vanishes for closed shells due to their stability, but also for half-filled shells, that are also particularly stable. iii) Apart from this, the EA is seen to increase as we fill a shell since along a row the increased nuclear charge tends to win over the added electron, hence causing a stabilization of orbitals. I would perhaps sharpen this discussion as well.

Response to the Reviewer:

In this section we simply wanted to make the statement that whereas the IEs are varying smoothly over the periodic table, the EAs show comparably much larger variations where some of them are easily explained, such as the increase in the EA as a shell is filled. Other features, such as the non-existence of a stable negative ion of nitrogen cannot be explained by a simple argument. We have rewritten these sentences following the advice from the referee, without getting into any details.

Suggested changes to the manuscript:

The sentences (page 3, line 67):

"The atomic IEs, which essentially are determined by the Coulomb attraction between the electrons and the nucleus, show a specific and well understood variation along the periodic table of elements. Starting from the lowest values

Suggested changes to the manuscript:

We have replaced the sentences (page 7, line 124):

"In At^-, the electron is detached from a p-state. Close to the threshold, the angular momentum of the outgoing electron will then be $l = 0$ due to the selection rules ($\Delta l = \pm 1$) and the centrifugal barrier preventing the emission of a d-wave electron ($l = 2$) [#]. The At^- ion is a closed shell system with no internal structure."

with:

"The ground state of At^- has a $6p^6\ ^1S_0$ configuration. Therefore, this state shows no term, fine or hyperfine structure splitting. Further, as for all other halogen negative ions, it is the only bound state. Hence, all At^- ions in the ion beam are in the same quantum state, and the relatively high temperature in the ion source does not give rise to internally excited ions. In the photodetachment process, the electron is detached from a p-state. Close to the threshold, the angular momentum of the outgoing electron will then be $l = 0$ due to the selection rules ($\Delta l = \pm 1$) and the centrifugal barrier preventing the emission of a d-wave electron ($l = 2$) [#]."

5

The manuscript concludes by stating that "the on-line technique presented in this work enables further EA measurements of artificially produced, short-lived radioactive elements with high precision" and "paving the way for both experimental and theoretical studies of superheavy elements". These are very interesting and promising perspectives indeed. However, in the present work the half-life of the species was around seven hours. Maybe the authors could discuss approximately what half-lives would be too short for the present setup?

Response to the Reviewer:

In terms of half-life of the investigated ion with the method used here, the limitation is the extraction and ionization from the target and ion source unit at ISOLDE, which has delivered ions with half-lifes in millisecond range.

Suggested changes to the manuscript:

We add after the sentence (page 14, line 266): "Finally, the on-line technique presented in this work enables further EA measurements of artificially produced, short-lived radioactive elements with high precision."
the sentence

"At ISOLDE, we can study isotopes with half-lifes down to the millisecond range, which is limited by the time needed to extract and transport the ions from the target unit to the GANDALPH detector."

in the lower left corner at the heaviest alkalines, a mostly steady trend towards higher values is observed both towards lighter elements with similar chemical behaviour in one column and along rows to the right side of the chart with halogens and noble gases, with only few exceptions. Conversely, the EAs display comparably strong irregularities and variations across the periodic table, as shown in Fig. 1. A number of elements such as all the noble gases do not form stable negative ions at all, and thus have negative EAs."

were replaced by the sentences:

"The atomic IEs show a highly regular variation along the periodic table of elements. Starting from lowest values in the lower left corner at the heaviest alkalines, a mostly steady trend towards higher values is observed both towards lighter elements with similar chemical behaviour in one column and along rows to the right side of the chart with halogenes and noble gases, with only few exceptions. Conversely, the EAs display comparably larger variations across the periodic table, as shown in Fig. 1. In this figure, some general features can be noted. For instance, the EA tends to increase as a shell is filled, then drops dramatically for elements with closed shell atomic structures, such as the noble gases, which do not form stable negative ions at all, and thus have negative EAs."

4 and 6

The measurement is based on the developed technique of laser-photodetachment. We learn that the ions are generated at high temperature, so an obvious thing we would like to know is to what extent they are cooled upon entering the spectrometer. Are we guaranteed that they are all in the ground state ? I believe the experimental setup is sound, but I feel this point should be sharpened.

On page 6 we read that the astatine anion "is a closed shell system with no internal structure". I think that the authors want to say that it does not contribute to any splittings of spectral lines. However, it sounds a bit awkward to say that such a multi-electron atom has no internal structure and I would recommend to reformulate.

Response to the reviewer:

We understand from comments 4 and 6 that we have been unclear in our description of the energy levels of At^- where we state that "The At^- ion is a closed shell system with no internal structure." What we meant to express here, is that there is only a single state, the ground state, that is bound. Further, this state has no term-, fine structure or hyperfine structure splittings. This means that all At^- ions are in a single quantum state. Hence, there is no Boltzman distribution among internal states, and the temperature in the ion source does not affect the experiment. We have rephrased to clarify.

7

Page 7, Figure 3: Show an expansion of the plot in the threshold region, 2.4155-2.4160 eV, with the assigned threshold clearly indicated, this to allow the reader to directly assess the accuracy of the determination. Also specify the energy separation between the last point before and the first point after the photodissociation onset; this energy resolution of the experiment is important.

Response to the Reviewer:

The distance between the data points, which corresponds to the step size of the wavelength of the laser, is about 100 micro eV. This can be seen more clearly in the inset of figure 3 that we have added.

Suggested changes to the manuscript:

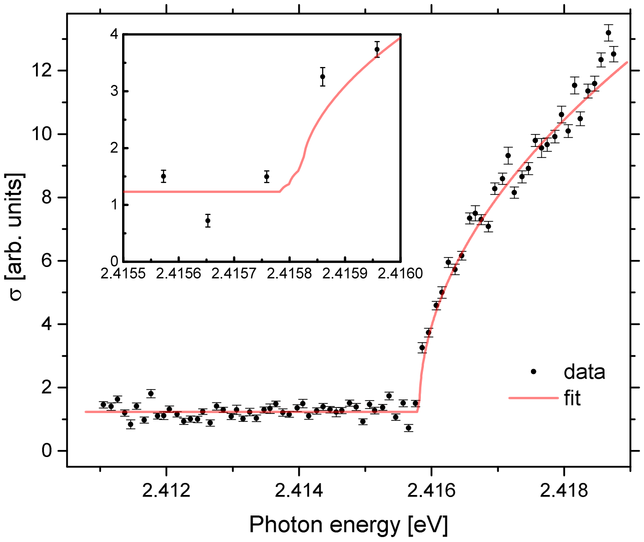

Figure 1: Threshold scan of the photodetachment of astatine. Neutralization cross section is measured as a function of the photon energy. The data points are the experimental measurements with one standard error represented with error bars, and the solid line is a fit of Eq. 1. The onset corresponds to the EA of ^{211}At. The inset shows the region around threshold, where the hyperfine levels of the ground state of the neutral atom are represented in the fit function.

We have added an inset in Fig. 3. and replaced the caption:

"Threshold scan of the photodetachment of astatine. Neutralization cross section is measured as a function of the photon energy. The data points are the experimental measurements with one standard error represented with error bars, and the solid line is a fit of Eq. 1. The onset corresponds to the EA of ^{211}At."

with:

"Threshold scan of the photodetachment of astatine. Neutralization cross section is measured as a function of the photon energy. The data points are the experimental measurements with one standard error represented with error bars, and the solid line is a fit of Eq. 1. The onset corresponds to the EA of ^{211}At. The inset shows the region around threshold, where the different onsets in the fit function represent the detachment to the hyperfine levels of the ground state of the neutral atom."

8

The computational machinery is essentially the same as reported by many of the same theoretician in PRL 118, 023002 (2017), but contrary to the EA/IE of the neutral gold atom the effect of adding higher excitations (T and Q) are quite modest, so that statement in the abstract "require incorporation of the electron-electron effects on the highest possible level" is perhaps a bit exaggerated. Please sharpen. Clearly very high-level calculations were carried out (provided a good choice of basis set was made).

Response to the Reviewer:

Indeed, the higher order effects for these atoms are less significant than for gold, but still non-negligible and important to include when attempting to achieve benchmark level accuracy. We have rephrased the sentence in the abstract.

Suggested changes to the manuscript:

The following sentence (page 2, line 38):

"This result is compared to state-of-the-art relativistic quantum mechanical calculations, which require incorporation of the electron-electron correlation effects on the highest level that can be currently achieved for many electron systems."

was replaced by:

"This result is compared to state-of-the-art relativistic quantum mechanical calculations that incorporate both the Breit and the QED corrections and the electron-electron correlation effects on the highest level that can be currently achieved for many electron systems."

9

In the conclusion: "in contrary to" ⟶ "contrary to"

Response to the Reviewer:

Suggested changes to the manuscript:

As suggested by reviewer, (page 13, line 252) "in contrary to" was changed to "contrary to".

10

In computational details: "is deemed" ⟶ "was deemed"

Response to the Reviewer:

Suggested changes to the manuscript:

As suggested by reviewer, (page 18, line 377) "is deemed" was changed to "was deemed".

11

The title of ref. 56 is very wrong ! There are also minor corrections to be done in the titles of refs. 7, 10 and 54.

Response to the Reviewer:

We would like to thank the reviewer for pointing out these typos.

Suggested changes to the manuscript:

Title of ref.56 (now ref. 59) was changed to "Dirac–Fock Atomic Electronic structure calculations using different nuclear charge distributions"
ref 7 (now 8): changed th to Th
ref 10 (now 12): "-" was added in the title
ref 54 (now 57): "-" was added (Hartree-Fock instead of Hartree Fock)

Response to reviewer 2

April 2020

Reviewer 2

This manuscript by Leimbach et al. reports the first experimental determination of the electron affinity (EA) of astatine using an elegant approach at CERN-ISOLDE. As the authors point out, EA is among the most fundamental atomic properties, and it can afford insights into elemental chemistry. The experimental result also provides a basis to assess computational results and methodologies. A new computed EA(At) is presented and compared with both the experimental and previous computational results. The impressive success of the experiment provides a platform for devising approaches to determine EAs of even heavier and more scarce elements. Both the experimental approach and the resulting determination of EA(At) merit publication in Nature Communications. The manuscript is generally well written and clear. I offer below some comments that need to be addressed by the authors when preparing a revision.

Author response:

We thank all the reviewers for the support, the time spent on the comments and consequently the valuable suggestions leading to the improvement of the manuscript. We thank the reviewer for the very constructive and thorough work. Additionally, the changes suggested by other reviewers are highlighted in the revised version of the manuscript.

1

Page 2, line 39: Change "electron-electron correlation effects on the highest possible level" to "electron-electron correlation effects on the currently highest practical level". Let's hope that in future even more accurate approaches are developed

Response to the Reviewer:

We fully agree with the reviewer.

Suggested changes to the manuscript:

The phrase (page 2, line 40) "electron-electron correlation effects on the highest possible level" was changed to "the highest level that can be currently achieved for many-electron systems"

2

Page 3, line 55: Provide a reference for the statement that "EA is mainly determined by electron-electron correlation". Given that EA is generally higher for small atoms it would be helpful to clarify the seeming implication here that electron-nucleus interactions have only minor effects on EA.

Response to the Reviewer:

This sentence was also commented on by referee 1. What we would like to express here is that the electron-electron correlation, due to the lack of the long range Couloumb interaction, is of greater importance in negative ions as compared with atoms or positive ions. We have therefore changed this sentence to make a correct statement, and also added a reference to a review on negative ions [Pegg2004].

Suggested changes to the manuscript:

The sentence (page 3, line 55) "Since the attraction from the nucleus is efficiently screened by the core electrons, the value of the EA is mainly determined by electron-electron correlation" has been replaced by: "Due to the lack of the long range Coloumb interaction, the electron-electron correlation is of greater importance in negative ions as compared with atoms or positive ions [#]."

3

Page 3, line 75: Change "largest EA" to "largest known EA".

Response to the Reviewer:

We agree with this suggestion.

Suggested changes to the manuscript:

As suggested by reviewer (page 3, line 79), "largest EA" was changed to "largest known EA".

4

Page 4, line 85: The EA of an element does not provide "precise knowledge about the chemical properties". At best it provides an imprecise basis to infer some chemical properties. Actual chemical properties of the likes of At are instead provided by approaches such as the elegant work described in ref. 38. Several of my following comments focus on the overemphasis on purported accurate derivation of condensed phase chemical behavior from EA, IE, and derived atomic properties.

Response to the Reviewer:

According to the comment, we have rephrased the statement.

Suggested changes to the manuscript:

The sentence (page 4, line 87) "Hence, an experimental determination of EA(At) is of fundamental interest, both to test sophisticated atomic theories and to gain precise knowledge about the chemical properties of this element."

has been replaced by:

"Hence, an experimental determination of EA(At) is of fundamental interest, both to test sophisticated atomic theories and to gain bases for inferring some chemical properties of this element."

5

Page 5, line 98: The EA is neither required nor sufficient to "describe these reaction kinetics" or "the stability of astatine compounds". Such claims as to the utility of EA for predicting or understanding complex condensed phase phenomena must be tempered

Response to the Reviewer:

According to this comment, the pointed out sentence has been tempered and rephrased.

Suggested changes to the manuscript:

The sentence (page 5, line 102) "In order to describe these reaction kinetics as well as the stability of astatine compounds, knowledge of the electron binding energy of the atomic anion, i.e. the EA, is required."

has been replaced by:

"The determination of the electron binding energy of the astatine anion, i.e. the EA, should help to better understand these reaction kinetics as well as the stability of involved astatine compounds."

6

Page 7, Figure 3: Show an expansion of the plot in the threshold region, 2.4155-2.4160 eV, with the assigned threshold clearly indicated, this to allow the reader to directly assess the accuracy of the determination. Also specify the energy separation between the last point before and the first point after the photodissociation onset; this energy resolution of the experiment is important.

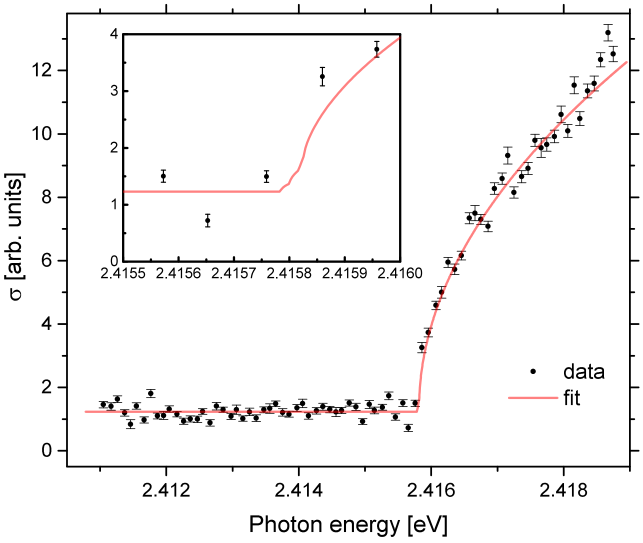

Figure 1: Threshold scan of the photodetachment of astatine. Neutralization cross section is measured as a function of the photon energy. The data points are the experimental measurements with one standard error represented with error bars, and the solid line is a fit of Eq. 1. The onset corresponds to the EA of ^{211}At. The inset shows the region around threshold, where the hyperfine levels of the ground state of the neutral atom are represented in the fit function.

Response to the Reviewer:

The distance between the data points, which corresponds to the step size of the wavelength of the laser, is approximately 100 micro eV. This can be seen more clearly now in the inset of figure 3, which we have added in the revised version.

Suggested changes to the manuscript:

We have added an inset in Fig. 3. and adapted the caption:

"Threshold scan of the photodetachment of astatine. Neutralization cross section is measured as a function of the photon energy. The data points are the experimental measurements with one standard error represented with error bars, and the solid line is a fit of Eq. 1. The onset corresponds to the EA of ^{211}At."

with:

"Threshold scan of the photodetachment of astatine. Neutralization cross section is measured as a function of the photon energy. The data points are the experimental measurements with one standard error represented with error bars, and the solid line is a fit of Eq. 1. The onset corresponds to the EA of ^{211}At. The inset shows the region around threshold, where the different onsets in the fit function represent the detachment to the hyperfine levels of the ground state of the neutral atom."

7

Page 8, line 132: It is interesting that the isotope 127I is specified. Were the computations for 211At? Is there a significant isotope variation of EA and derived atomic properties?

Response to the Reviewer:

The DIRAC program uses the mass of the most abundant isotope, i.e. 127 for iodine. In case of At, the 210 mass is used. However, the variation of atomic properties with isotope is negligible, and beyond the calculation precision. To avoid ambiguity, we remove the mass number.

Suggested changes to the manuscript:

Replace the sentence (page 9, line 156):

"Alongside the measurements, state-of-the-art calculations of the electron affinities of astatine and of its lighter homologue, iodine (^{127}I) were carried out."

with:

"Alongside the measurements, state-of-the-art calculations of the electron affinities of astatine and of its lighter homologue, iodine, were carried out."

8

Page 9, Discussion Section: Table 3 and discussion of those values belongs here rather than in the Methods section. In the first paragraph of this section it is noted that "our computed value is in excellent agreement with experiment" and

that the employed computational approach is "necessary for achieving benchmark accuracy". However, it is apparent from Table 3 that other computed values are also in very good agreement with the new experimental value, which does not per se validate those computational methodologies. The excellent agreement for the value from ref. 15, for example, neither certifies the MCDHF method nor its application to other systems. The current agreement does not validate the employed computational approach beyond its application to EA(At).

Response to the Reviewer:

We fully agree with the referee that Table 3 with discussion better fits in discussion section. We will move it if the editor allows us a slight extension of the length of the paper.

The employed method includes the different higher order computational effects in a systematic and balanced way, and, as such, should be valid for achieving high or benchmark accuracy for the majority of atoms where this method is applicable. The balanced methodology is exemplified by our result for EA(I) which achieved similar accuracy as our EA(At) result using the same methodology. This was not the case in any of the referenced works. Nonetheless, we have modified the sentence in point to soften the message.

Suggested changes to the manuscript:

We removed the section "Comparison to previous theoretical results." and moved Table 3 and text into the discussion section. Hence, we have removed the sentence:

"For a more detailed comparison of our computed results with previous theoretical investigations we refer the reader to the Methods section."

and replaced (page 10, line 181):

"This clearly demonstrates that careful, systematic, and as complete as possible inclusion of higher-order correlation and relativistic contributions is necessary for achieving benchmark accuracy."

with:

"This shows that careful, systematic, and as complete as possible inclusion of higher-order correlation and relativistic contributions makes it possible to achieve benchmark accuracy in atomic calculations."

9

Although the rationale on page 17 for the assigned uncertainty to the computed EA(At) is reasonable, any such assignments must be qualified by the acknowledgement of possible unrecognized errors in the computed values. The unreasonably (and evidently incorrect) small uncertainty assigned to the ref.

12 value in Table 3 clearly illustrates this common mistake that should not be repeated here despite the good agreement between the current experiment and theory.

Response to the Reviewer:

We aim to assign reasonable uncertainties that reliably reflect the actual accuracy of the method. Relativistic coupled cluster approach has the advantage of transparency, which gives us a very clear idea of the included (and the missing) effects. Nonetheless, there can occur unexpected computational errors, which we now refer to in the manuscript.

Suggested changes to the manuscript:

We have made slight modifications (highlighted in red) to the paragraph describing uncertainty evaluation (page 19, line 394):

"The three remaining known sources of error in these calculations are the basis set incompleteness, the neglect of even higher excitations beyond (Q), and the higher-order QED contributions. The first of these is the largest. We have extrapolated our results to the complete basis set limit, and as the associated error, we take half the difference between the CBS result and the doubly augmented ae4z (d-aug-ae4z) basis set value which is 0.015 eV. We assume that the effect of the higher excitations should not exceed the (Q) contribution of 0.004 eV, and that the error due to the incomplete treatment of the QED effects is not larger than the vacuum polarization and the self energy contributions of 0.003 eV. Combining the above sources of error and assuming them to be independent (and assuming no uncertainties beyond those discussed above), the total conservative uncertainty estimate on the calculated EA of At is 0.016 eV, dominated by the basis set effects."

10

Page 10, line 167: The sentence beginning with "In fact, the redox potential..." needs to be clearly and properly referenced and justified.

Response to the Reviewer:

We have made modifications to this sentence as stated below.

Suggested changes to the manuscript:

The pointed out sentence (page 11, line 203) "In fact, the redox potential associated with the formation of At− is primarily determined by its EA, and to a lesser extent by the difference of Gibbs' free energy of solvation between the anion and the neutral atom."

has been corrected as follows:
"The reduction potential associated with the formation of At− is determined in half by the EA. Indeed, the reduction potential in solution can be evaluated from a thermodynamic cycle involving the reduction reaction in the gas phase [39], whose Gibbs free energy essentially comes down to the electron affinity (only trifling contributions come from (i) the free energy of the gas-phase free electron [39], and (ii) the remaining entropic term resulting from the electronic partition function of At ($2P_{3/2}$), and the difference of Gibbs free energy of solvation between the anion and the neutral atom, which roughly accounts for 2.5eV ($\Delta S_{sol}(At^-) \approx -68$ kcal mol^{-1} according to a recent estimate [40], and solvation free energies of neutral solutes do not exceed few kcal mol^{-1} [39]).", adding references

[39]: C. J. Cramer, Essentials of Computational Chemistry: Theories and Models, J. Wiley and Sons, Chichester, 2nd edn, 2004.
and [40]: F. Réal et al., Structural, dynamical, and transport properties of the hydrated halides: How do At^- bulk properties compare with those of the other halides, from F^- to I^-?, J. Chem. Phys. 144, 124513 (2016)

11

Page 10, line 172: Although it may be valid that E°(At-/At+) "directly depends on the EA(At) and IE(At)" it should be emphasized that other factors are important. The role of solvation of At- and At+ are key aspects, as discussed in ref. 39.

Response to the Reviewer:

We agree with the comment and modified the sentence accordingly.

Suggested changes to the manuscript:

The pointed out sentence (page 11, line 211) "In addition to the EA, the IE contributes also to the determination of the nature of elemental forms of astatine in aqueous solutions: the Pourbaix (potential/pH) diagram of astatine shows coexistence of the At+ and At− ions, whose dominance domains are governed by the redox potential E°(At−/At+), which directly depends on the EA(At) and IE(At) [38,39]."
has been revised as follows:
"In addition to the EA, the IE contributes also to the determination of the nature of elemental forms of astatine in aqueous solutions: the Pourbaix (potential/pH) diagram of astatine shows coexistence of the At^+ and At^- ions. Their dominance domains are governed by the redox potential E°(At^-/At^+) [41], which can be evaluated as well from a thermodynamic cycle involving (i) the formation in gas phase of astatide from the At+ cation [42], whose reaction

free energy essentially comes down to the sum of EA(At) and IE(At), and (ii) the difference of solvation free energy between the anion and the cation.”

12

Page 11, Table 2: The key result of the work is the first experimental value for EA(At). The new computed value is so similar to all previous computed values (see Table 3) that it really provides no new chemical insights. The derived values in Table 2 are interesting but there is no significant revision in the predicted chemistry of At. The values from Geerlings and co-workers (ref. 47) should be included in Table 2 as they are actually remarkably good and they serve to predict essential chemical properties of At. For example, revision of the electronegativity of At from 5.74 eV in ref. 47 to 5.86665(7) eV here does not significantly revise the predicted chemistry of At; nor does it significantly revise our understanding of the chemistry of At.

Response to the Reviewer:

We agree with the referee that the theoretical results of Geerlings and co-workers are impressive. However, table 2 offers an update on the recommended values with the currently highest possible accuracy resulting from high precision measurements.
We therefore prefer to not include Geerlings and co-workers in table 2 since that would additionally lead to extended discussions, such as why their data of the IP of 9.04 eV deviates from the experimental value of 9.31751(8)eV.
In order to clarify this point, we have changed the sentences as described below.

Suggested changes to the manuscript:

We have changed the sentence (page 13, line 239):

”The list of chemical descriptors presented in Tab. 3 represents a significant advance over the computed data reported by Paul Geerlings and co-workers [43], and may be regarded as basic properties which will serve as the foundation for the design and the assessment of innovative astatine radiopharmaceuticals by theoretical and experimental chemists.”

to:

“The list of chemical descriptors presented in Tab. 3 represents an advance over the computed data reported by Paul Geerlings and co-workers [43] by deriving them from high precision measurements, and may be regarded as basic properties which will serve as the foundation for the design and the assessment of innovative astatine radiopharmaceuticals by theoretical and experimental chemists.”

13

Page 12, line 216: The sentence beginning "Hence, the new information..." should be deleted or revised. There is no new information about astatine's chemical properties that will guide development of innovative radio-labelling protocols. Instead there is some refinement in parameters that might have been employed to roughly predict some aspects of the chemical behavior of At, if this behavior were not already known.

Response to the Reviewer:

We have changed this sentence according to the suggestion by the reviewer.

Suggested changes to the manuscript:

The sentence (page 14, line 260) "Hence, the new information about astatine's chemical properties presented here will be of great importance in the development of innovative radio-labelling protocols."
has been replaced by:
"Hence, accurate values of electron affinity, electronegativity, softness and electrophilicity, all issued from experiments, open up several perspectives that chemists and radiopharmacists can take advantage to understand the stability of astatine-labelled compounds. Considering that oxidative mechanisms may be responsible for in vivo dehalogenation [#], the expected polarization towards the carbon atom, at the expense of astatine, has notably been highlighted for At-C chemical bonds. Potential impacts on the development of more efficient radio-labelling protocols cannot be ruled out.".

14

Page 12, line 219: The point in the sentence beginning with "Finally,..." is a very positive result of this work. The manuscript would benefit from elaboration as to how the technique might be modified for application to much lower ion abundances such as would be the case for superheavy elements.

Response to the Reviewer:

Indeed, studies of e.g. trans-uranium elements will give much lower yields, which required an improved experimental technique. We are currently developing such a technique where the GANDALPH detector will be replaced with a MR-TOF system where produced ions will be allowed to interact with the laser light for a much longer time.

Suggested changes to the manuscript:

We have after the sentences starting ”Finally, ..” added the sentences (page 14, line 270): ”However, studies of the short-lived elements which are normally produced with lower yields will require an improved detection system. We are currently developing a new detector based on the Multiple Reflection Time-of-Flight (MR-TOF) technique where each produced ion will be allowed to interact with the laser light for a much longer time.”

15

Page 12, line 220: Regarding the sentence beginning with “Furthermore. . . ”, favorable results for At (Z = 85) do not demonstrate nor pave the way for theoretical studies of superheavy elements ($Z \geq 104$). Emphasize here and elsewhere the impressive experimental “paving”.

Response to the Reviewer:

We have rewritten the last sentence of this section.

Suggested changes to the manuscript:

The final sentence of this paragraph (page 14, line 273) is replaced with:

”Furthermore, the excellent performance of the relativistic coupled cluster for astatine, and the robust scheme for estimation of theoretical uncertainties demonstrates the strong predictive power of this method. This will become extremely important for the superheavy elements where the low production rates and short lifetimes will necessitate reliable theoretical support for the success of the measurements. ”

Response to reviewer 3

April 2020

Reviewer 3

Report on "The electron affinity of astatine", ms. NCOMMS-20-05667-T by David Leimbach et al. This manuscript reports on the first experimental measurement of the electron affinity of astatine, with a comparison with ab initio calculations. The work seems basically sound and it will very likely make a valuable contribution to our knowledge of atomic electron affinities. A few details however call for additional explanations or amendments.

Author response:

We thank all the reviewers for the support, the time spent on the comments and consequently the valuable suggestions leading to the improvement of the manuscript.

We have addressed the issues raised point by point in the text below, indicate the resulting change to the manuscript, hopefully giving the requested explanations and suggested amendments by Reviewer 3.

Additionally, the changes suggested by other reviewers are highlighted in the revised version of the manuscript.

1

Figure 1 should be updated taking the publication of a recent measurement of the electron affinities of Pr, Nd and Tb into account (the electron affinity of Pr had been in principle already known but the measurement led to a substantial reduction of its value): X. Fu et al., Phys. Rev. A 101 (2020) 022502.

Response to the Reviewer:

We thank the reviewer to point out these recently published values.

Suggested changes to the manuscript:

Figure 1 was updated and references to X. Fu et al. added in the caption.

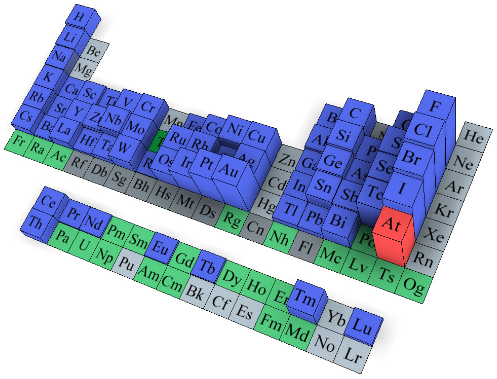

Figure 1: Electron affinities across the periodic table. The height corresponds to the measured value of the electron affinity of the corresponding element [#]. Astatine is highlighted in red. Blue indicates elements that are experimentally determined to have a positive EA, i.e. to form stable negative ions. Elements that are predicted to form stable negative ions but not yet have been experimentally investigated are indicated in green, while those in light grey are predicted to not form a stable negative ion, i.e. have an negative EA. Finally, elements that neither have been experimentally observed nor investigated theoretically, are indicated with dark grey.

2

On page 6 I do not understand why so many electrons appear in the zoomed parts of figure 2, especially zoom number 2: why should photodetachment make two electrons appear?

Response to the Reviewer:

The second electron was intended to show that only one electron is detached. We agree with the reviewer that this is unclear and have therefore removed the second electron in the figure.

Suggested changes to the manuscript:

As described above.

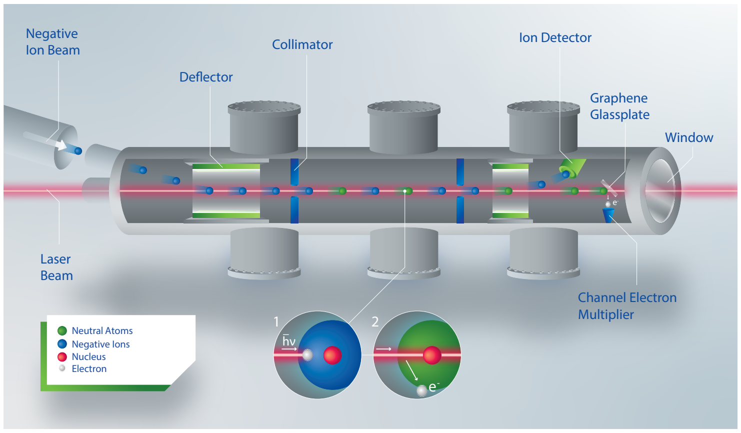

Figure 2: Schematic diagram of the experimental setup. From left to right: A beam of negative astatine ions is guided into the Gothenburg ANion Detector for Affinity measurements by Laser PHotodetachment (GANDALPH)[#], where the ion beam is overlapped with a frequency tuneable laser beam in the interaction region in either co- or counter-propagating geometry. By absorbing a photon (Inset 1), an electron can gain enough energy to be ejected from the ion, thereby creating a neutral atom (Inset 2). After the interaction region, the charged particles are deflected into an ion detector, while neutralized atoms continue moving straight to the graphene-coated glass plate downstream and create secondary electrons, which are detected by a channel electron multiplier[#].

3

Page 7, “Heaviside” is Oliver Heaviside’s name and thus deserves an initial capital letter

Response to the Reviewer:

We thank the reviewer for pointing out this typo.

Suggested changes to the manuscript:

(page 7, line 138) ”Heaviside” instead of ”heaviside”.

4

On page 8 I wish we were given the results of the “reference measurements” performed on 127I to consolidate the estimation of the uncertainty. The result of the calculation of the electron affinity of iodine is given explicitly; why not show us the measured electron affinity?

Response to the Reviewer:

The reference measurement on iodine was performed in order to check the stability of the system since a drift in the energy of the ions would change the measured threshold value. However, the reference measurement was only performed using counter-propagating beams, and could hence not be used to deduce an absolute threshold value.

Suggested changes to the manuscript:

To clarify we have changed the sentence (page 7, line 149):

"The combined systematic error of photon energy and beam energy is estimated to be smaller than 20 µeV by comparing two reference measurements of stable ^{127}I which were performed before and after the experiment on astatine, under the same experimental conditions."

with:

"We measured the threshold for the stable ^{127}I before and after the experiment on astatine, under the same experimental conditions. Those measurement differed with less than 20 µeV, This gives an estimate of a systematic error in instabilities in the photon energy determination and ion beam energy stability."

5

It is not clear whether the described theoretical calculation of the electron affinity does include the hyperfine splitting of the neutral atom. My impression, even though the authors tell the nuclear volume is taken into account, is that the splitting is not considered. As a consequence, the final value of the calculated electron affinity is probably the hyperfine-averaged value, while the experimental value refers to the actual ground level of the neutral atom, i.e. the lowest hyperfine sublevel. The difference, if I translate Cubiss et al.'s parameters properly, is a bit more than 10 µeV. This is much smaller than the uncertainty of the calculated value, but this is enough to change the last digit of the experimental value, if one wants to translate it into a hyperfine-averaged one. The question of whether the hyperfine structure has been included in the calculation should be made clear anyway and, if comparisons are made, those comparisons must be made between identically defined quantities.

Response to the Reviewer:

The calculations did not include hyperfine structure. We have amended the text to indicate this explicitly.

Suggested changes to the manuscript:

The following sentence was added at the end of the "Computational details" section (page 19, line 403):

"It should be noted that the hyperfine structure of the neutral atom was not considered in the calculations. However, the correction due the hyperfine structure would be of the order of 10 μeV, to be compared with the estimated uncertainty in the calculation of 1600 μeV. Hence, correcting for the hyperfine structure would not change the given theoretical value and can therefore be neglected."

6

On page 9, the authors tell us of a "meV accuracy", but the quoted reference actually tells of a 1.4 meV difference between theory and experiment, which is slightly larger. The accuracy reached by previous calculations on gold should be described in a more precise way, without overstating the accuracy reached by reference 35.

Response to the Reviewer:

The phrasing was changed according to the suggestion of the reviewer.

Suggested changes to the manuscript:

The phrasing (page 9, line 170) was changed to "an accuracy of 1.4meV".

7

On page 10, the authors wonder that At has the lowest electron affinity among the halogens. This was, however, quite predictable as a fine structure effect. Even though the fine structure of the ground term of astatine has remained, as far as I know, unmeasured, one can rely on the observation of a very large fine structure in iodine (about 0.94 eV) to predict that the fine structure splitting of neutral astatine, the larger Z the greater the fine structure, will be even greater than 1 eV. As the negative ion At-, due to its complete outer shell, has no fine structure, huge fine structure lowering of the atom's ground level has the straightforward effect of making the electron affinity of At substantially smaller than the electron affinities of lighter atoms in the same column. A similar effect has already been observed in Pb, which is not that different from the lighter atoms of its column IV B, but owes its much smaller electron affinity to a huge fine structure splitting of the neutral ground level (and, again, absence of ground-term fine structure splitting, which, for the anions of column IV B, is due to the fact that this ground term is an S one), see D. Bresteau et al., J. Phys. B: At. Mol. Opt. Phys. 52 (2019) 065001.

Response to the Reviewer:

We agree that there is no surprise in finding that the the EA of astatine is smaller than the lighter halogens and have therefore modified our statement.

Suggested changes to the manuscript:

We have changed the sentence (page 10, line 198):
"Our result of the EA of astatine, 2.415 78(7) eV, indicates that among the halogen elements, astatine has the lowest EA."

to:
"Our result of the EA of astatine, 2.415 78(7) eV, indicates, as expected, that among the naturally occurring halogen elements, astatine has the lowest EA."

Final comment of reviewer 3

These considerations would deserve being alluded to, especially when one compares the electron affinity of astatine to the electron affinities of the other atoms of column VII B, and of course made more precise if the fine structure of astatine happens to have been measured somewhere, which I have not been aware of.

Response to the Reviewer:

We agree and have hence followed the detailed advises of the referee.

Reviewers' Comments:

Reviewer #1:
Remarks to the Author:
The revised manuscript is clearly improved, and with the missing computational details in place, I am reassured that the theoretical calculations are of excellent quality. There are, however, some formulations that need sharpening and also what I call "stupid errors", errors that could have been easily been spotted by a bit more careful reading of the manuscript. The latter I find disappointing in view of the standards of the journal to which the manuscript has been submitted. I recommend that the manuscript after the following minor revisions:

1) In the first paragraph of the introduction we now find the sentence: "Due to the lack of the long range Coulomb interaction, the electron-electron correlation is of greater importance in negative ions as compared with atoms or positive ions" for which a 2004 paper by Pegg is cited. However, as it stands this phrase is not easily understandable. Just thinking about the Hamiltonian describing the system, long-range Coulomb forces are there. Going to the reference we find: "The structure of a negative ion is qualitatively different from that of an atom or positive ion. This difference can be traced to the nature of the force that binds the outermost electron. In atoms and positive ions the valence electron moves asymptotically in the long-range Coulomb field of the positively charged core. The relatively strong force creates a 1/r potential that is capable of supporting an infinite spectrum of bound states that converge on the ionization limit. In contrast, the outermost electron in a negative ion experiences a much weaker and short-range induced force that arises from the polarization of the atomic core." I think what the authors are trying to say is that the since the excess electron asymptotically sees a neutral system, electron affinities become more sensitive to electron correlation. Please sharpen.
2) Figure numbers are missing throughout the text !
3) At the bottom of page 8 we read: "The reduction potential associated with the formation of At- is determined in half by the EA." It is not clear to me what is meantby "in half". Please sharpen. Next comes a VERY long and rambling sentence. Please reformulate in short and clear sentences.
4) In the sentence on p15: "modelled by a Gaussian charge distribution within the DIRAC15 program package" it is more clear if Ref. 58 is moved back to Gaussian charge distribution.
5) Carefully check author names in Refs. 39 and 47.
6) I guess gandalph should be in capital letters in Ref. 50.
7) In Table 2 we find for method 6 the acronym QEDD. Is there a 'D" too many ?

Language issues:
- p3: "occurring elements in the halogen" -> "occurring element in the halogen"
- p6: "differed with less" -> "differed by less"
- p7: "powerful for treatment" -> "powerful for the treatment"
- p29: Caption Fig 3: "Neutralization cross section is measured" -> "The neutralization cross section is measured"

Reviewer #2:
Remarks to the Author:
The authors have effectively addressed my comments in the revised version of this manuscript, which I recommend for publication in Nature Communications.

Reviewer #3:
Remarks to the Author:
Second report on "The electron affinity of astatine", ms. NCOMMS-20-05667A by David Leimbach et al.

As for a majority of remarks made by the referees, the manuscript appears to have been corrected in a satisfactory way. One exception is figure 1, which has not yet been corrected to take into account the measurement of the electron affinities of Pr, Nd and Tb by Fu et al. [67]. This must be done, imperatively. The fact that an actually updated figure appears in the authors' response to referees demonstrates that this should not be too difficult, even though the fact appears particularly misleading.

A few other details still call for some revisions:

- Figures are not numbered when called in the text (there is a blank instead). I thus cannot check whether the calls for figures are numbered appropriately and will thus decline any responsibility relative to figure numbering.
- Line 592: "the design of Fig. and ." apparently even contains more blanks...
- Line 295: "particless-1" probably stands for "particles•s-1", please check that this unit be properly printed or, to make it surer, have it printed "particles/s".
- Lines 405 and 408: the uncertainty of the calculation is told to be 0.016 eV, then 1600 μeV. These are not equal quantities.
- Line 503: "how do at bulk" should be printed "how do At- bulk", please restore the capital letter and minus sign.
- Line 534: "photodetachment of radioactive 128I" was actually "photodetachment of radioactive 128I-", please restore the – exponent.
- Line 536: "gandalph", up to that point, has been printed in capital letters, please homogenise.
- Line 585: I have up to now not been aware that we had several "European Unions", one is already difficult enough to manage...
- Table 2: the experimental electron affinity of At suddenly gets an improved precision, 2.41578(5) instead of 2.41578(7) eV, as in the rest of the paper. Please explain or correct.

EA of At response to reviewers 2

July 3, 2020

We would like to thank all reviewers again for their thorough work leading to an improvement of our manuscript.

Reviewer 1

1

In the first paragraph of the introduction we now find the sentence: "Due to the lack of the long range Coulomb interaction, the electron-electron correlation is of greater importance in negative ions as compared with atoms or positive ions" for which a 2004 paper by Pegg is cited. However, as it stands this phrase is not easily understandable. Just thinking about the Hamiltonian describing the system, long-range Coulomb forces are there. Going to the reference we find: "The structure of a negative ion is qualitatively different from that of an atom or positive ion. This difference can be traced to the nature of the force that binds the outermost electron. In atoms and positive ions the valence electron moves asymptotically in the long-range Coulomb field of the positively charged core. The relatively strong force creates a 1/r potential that is capable of supporting an infinite spectrum of bound states that converge on the ionization limit. In contrast, the outermost electron in a negative ion experiences a much weaker and short-range induced force that arises from the polarization of the atomic core." I think what the authors are trying to say is that the since the excess electron asymptotically sees a neutral system, electron affinities become more sensitive to electron correlation. Please sharpen.

Response to the Reviewer:

We have change the text according to the advice of the referee.

Suggested changes to the manuscript:

We have replaced the sentence with "Contrary to neutral atoms or positive ions, the excess electron in a negative ion asymptotically sees a neutral system. As a consequence, the electron-electron correlation plays a very important role

in various properties of the negative ions, and in particular in their electron affinities[Pegg2004]."

2

Figure numbers are missing throughout the text !

Response to the Reviewer:

The figure numbers now appear in the appropriate positions.

Suggested changes to the manuscript:

3

At the bottom of page 8 we read: "The reduction potential associated with the formation of At- is determined in half by the EA." It is not clear to me what is meant by "in half". Please sharpen. Next comes a VERY long and rambling sentence. Please reformulate in short and clear sentences.

Response to the Reviewer:

As suggested by the reviewer, we have rephrased the paragraph as stated below to clarify.

Suggested changes to the manuscript:

The mentioned paragraph was changed to: "A significant part of the value of the reduction potential associated with the formation of At^- comes from the EA. Indeed, the reduction potential in solution can be evaluated from a thermodynamic cycle involving (i) the reduction reaction in the gas phase[Cramer2004], and (ii) the difference of Gibbs free energy of solvation between the anion and the neutral atom. The Gibbs free energy corresponding to (i) essentially comes down to the electron affinity, since the electronic partition function of At ($^2P_{3/2}$) yields a trifling contribution and the free energy of the gas-phase free electron is almost null[Cramer2004]. The contribution of (ii) is similar to (i), $\approx$2.5 eV, since the solvation free energies of neutral solutes do not exceed few kcal per mol[Cramer2004] and, according to a recent estimate[doi:10.1063/1.4944613], $\Delta G_{sol}(At^-) \approx -68$ kcal/mol. In addition to the EA, the IE also contributes to the determination of the nature of elemental forms of astatine in aqueous solutions: the Pourbaix (potential/pH) diagram of astatine shows coexistence of the At^+ and At^- ions."

4

In the sentence on p15: "modelled by a Gaussian charge distribution within the DIRAC15 program package" it is more clear if Ref. 58 is moved back to Gaussian charge distribution.

Response to the Reviewer:

The citation was moved as suggested by the reviewer.

Suggested changes to the manuscript:

5

Carefully check author names in Refs. 39 and 47

Response to the Reviewer:

Author names in Ref. 39 were changed to Giju, K.T. Geerlings, P. and in Ref. 47. to Vosicki, B., Björnstad, T., Carraz, L. C., Heinemeier, J. Ravn, H.L.

Suggested changes to the manuscript:

6

I guess gandalph should be in capital letters in Ref. 50.

Response to the Reviewer:

We have ensured the capitalization of GANDALPH.

Suggested changes to the manuscript:

7

In Table 2 we find for method 6 the acronym QEDD. Is there a 'D" too many ?

Response to the Reviewer:

We thank the reviewer for pointing out this typo. We have removed the additional D.

Suggested changes to the manuscript:

Language issues

- p3: "occurring elements in the halogen" to "occurring element in the halogen"
- p6: "differed with less" to "differed by less" 7: "powerful for treatment" to "powerful for the treatment"
- p29: Caption Fig 3: "Neutralization cross section is measured" to "The neutralization cross section is measured"

Response to the Reviewer:

All language issues raised by the reviewer have been addressed according to the suggestions.

Suggested changes to the manuscript:

Reviewer 2

The authors have effectively addressed my comments in the revised version of this manuscript, which I recommend for publication in Nature Communications.

Response to the Reviewer:

We thank the reviewer again for the work leading to the improvement of our manuscript.

Reviewer 3

1

One exception is figure 1, which has not yet been corrected to take into account the measurement of the electron affinities of Pr, Nd and Tb by Fu et al. [67]. This must be done, imperatively. The fact that an actually updated figure appears in the authors' response to referees demonstrates that this should not be too difficult, even though the fact appears particularly misleading.

Response to the Reviewer:

The correct figure is now displayed in the manuscript.

Suggested changes to the manuscript:

2

Figures are not numbered when called in the text (there is a blank instead). I thus cannot check whether the calls for figures are numbered appropriately and will thus decline any responsibility relative to figure numbering.

Response to the Reviewer:

Figure references are now displayed in the appropriate positions.

Suggested changes to the manuscript:

3

Line 592: “the design of Fig. and .” apparently even contains more blanks...

Response to the Reviewer:

Figures are now correctly referenced here as well.

Suggested changes to the manuscript:

4

Line 295: “particless-1” probably stands for “particles · s^{-1}”, please check that this unit be properly printed or, to make it surer, have it printed “particles/s”.

Response to the Reviewer:

We agree with the reviewer and have changed the unit to ”particles per s”.

5

Lines 405 and 408: the uncertainty of the calculation is told to be 0.016 eV, then 1600 µeV. These are not equal quantities.

Response to the Reviewer:

The uncertainty of the calculation was 0.016 eV, which is now consistent throughout the manuscript.

6

Line 503: “how do at bulk” should be printed “how do At- bulk”, please restore the capital letter and minus sign.

Response to the Reviewer:

The reference title is now correctly formatted.

7

Line 534: “photodetachment of radioactive 128I” was actually “photodetachment of radioactive 128I-”, please restore the – exponent.

Response to the Reviewer:

The minus sign was added.

8

Line 536: “gandalph”, up to that point, has been printed in capital letters, please homogenise.

Response to the Reviewer:

GANDALPH is now spelled correctly in capitalized letters.

9

Line 585: I have up to now not been aware that we had several “European Unions”, one is already difficult enough to manage...

Response to the Reviewer:

The s was removed.

10

Table 2: the experimental electron affinity of At suddenly gets an improved precision, 2.41578(5) instead of 2.41578(7) eV, as in the rest of the paper. Please explain or correct.

Response to the Reviewer:

We have corrected this typo to be consistent with our result of 2.41578(7) eV.
